# Novel Method for Vibration Sensor-Based Instantaneous Defect Frequency Estimation for Rolling Bearings Under Non-Stationary Conditions

**DOI:** 10.3390/s20185201

**Published:** 2020-09-11

**Authors:** Dezun Zhao, Len Gelman, Fulei Chu, Andrew Ball

**Affiliations:** 1 Centre for Efficiency and Performance Engineering (CEPE), School of Computing and Engineering, University of Huddersfield, Huddersfield HD1 3DH, UK; D.Zhao@hud.ac.uk (D.Z.); L.Gelman@hud.ac.uk (L.G.); 2 Department of Mechanical Engineering, Tsinghua University, Beijing 100084, China; chufl@mail.tsinghua.edu.cn

**Keywords:** vibration sensor-based fault diagnosis, digital signal processing

## Abstract

It is proposed a novel instantaneous frequency estimation technology, multi-generalized demodulation transform, for non-stationary signals, whose true time variations of instantaneous frequencies are unknown and difficult to extract from the time-frequency representation due to essentially noisy environment. Theoretical bases of the novel instantaneous frequency estimation technology are created. The main innovations are summarized as: (a) novel instantaneous frequency estimation technology, multi-generalized demodulation transform, is proposed, (b) novel instantaneous frequency estimation results, obtained by simulation, for four types of amplitude and frequency modulated non-stationary single and multicomponent signals under strong background noise (signal to noise ratio is −5 dB), and (c) novel experimental instantaneous frequency estimation results for defect frequency of rolling bearings for multiple defect frequency harmonics, using the proposed technology in non-stationary conditions and in conditions of different levels of noise interference, including a strong noise interference. Quantitative instantaneous frequency estimation errors are employed to evaluate performance of the proposed IF estimation technology. Simulation and experimental estimation results show high effectiveness of the proposed estimation technology.

## 1. Introduction

Non-stationary signals occur in many fields, such as radar system, sonar, speech and mechanical systems [1]. Effective nonstationary signal processing methods are important for industrial applications. In recent years, scholars have developed some effective methods to characterize time-varying signal properties.

Time-frequency analysis (TFA) [2] is an effective method to display time-varying instantaneous frequencies (IFs) and time-varying instantaneous amplitudes of nonstationary signal. The traditional TFA methods include linear TFA such as the short-time Fourier transform (STFT), the wavelet transform (WT), and the bilinear/quadratic TFA such as the Wigner Ville distribution (WVD). However, these methods are restricted by the Heisenberg uncertainty principle and cross terms. Time frequency representation (TFR) with insufficient time-frequency resolutions are not providing accurate estimations of nonstationary signal, which essentially restrict their engineering applications. For improving the time-frequency resolutions, the time-frequency reassignment methods are developed, e.g., the synchrosqueezing transform (SST), demodulated SST and synchro-extracting transform (SET) [3]. The reassignment methods reduce energy spread via assigning the average of energy in a domain to the gravity center of these energy contributions.

It is well known that synchrosqueezed time–frequency representations are concentrated on an IF. However, it is also known that [4] synchrosqueezed time–frequency representations do not provide better time–frequency resolutions for components that have high time variability, as compared to the original STFT or the WT. The synchrosqueezing just sums all the interferences, presented in the original STFT and the WT, into a more compact frequency region. However, even if the components appear to be more compact, as a result of synchrosqueezed time–frequency representations, this does not mean, that their parameters can be better evaluated. The SST is similar to the original STFT and the WT and, although being perfectly concentrated, does not necessarily provide better resolution properties than the respective original STFT and WT.

Therefore, the “correct” amplitude peaks cannot be identified more easily in SST than in the original STFT and WT. This is due to a serious drawback of synchrosqueezed representations in that, in contrast to the STFT and WT, the peak amplitudes in the synchrosqueezed time–frequency representations are not proportional to the amplitudes of the corresponding spectral component; instead, they are largely determined by the parameters of the used frequency discretization. Therefore, it is known that, even if one estimated component has a smaller amplitude than another, it may still have a much higher peak in the synchrosqueezed time–frequency representations.

Generally, the relationships between the peak amplitudes of synchrosqueezed representation amplitudes of the corresponding spectral component depend on the frequency discretization and these relationships are nonlinear, time-varying, and are dependent on many factors, e.g., possible amplitude and frequency modulation of the component and noise. Hence, the outcomes of IF estimation by SST will also depend on the frequency discretization, amplitude modulation (AM), frequency modulation (FM), and the presence of noise.

This effect is additionally aggravated by the following factor: due to the non-smoothness of the SST, it is hard to apply peak interpolation, so, amplitude peaks remain discrete, and this amplitude discretization also affects the performance of IF evaluation. Due to the above factors, employing SST peak amplitudes for IF estimation can lead to unpredictable unstable results.

Finally, if the SST is used as the postprocessing technique of the STFT, which is not able to efficiently analyze nonstationary signals, the energy concentration of SET spectra will be essentially influenced by time–frequency resolution drawbacks of the STFT, when processing data.

In order to overcome the drawbacks of the SST method and obtain a better time–frequency resolution for strong FM signals, various methods have been developed in recent decades. A generalized SST was proposed [5] by demodulating the time-varying signal into a purely harmonic version. An iterative generalized SST [6] was developed to address multi-component signals with distinct FM laws. A matching SST demodulated transform [7], based on the polynomial or the Fourier series, was proposed to match time-varying IF. For all the above demodulated methods, the exact time-varying FM dependency of the signal is needed in advance. However, due to the complexity of practice diagnosis cases, it is hard to estimate the exact demodulated parameters, especially for AM-FM signals [8].

A popular method is to use the high-order information of the signal, such as the chirp rate, or frequency acceleration, to enhance the energy concentration for strong FM, which involves second-order SST [9] and higher order SST [10]. However, higher-order SST techniques are easy to be affected by noise because they are computed by the high-order derivative of the STFT and the WT. Recently, a new multi-SST (MSST) technique [11] was presented, which employs an iterative reassignment procedure to concentrate the blurry energy in a step-wise manner. MSST squeezes the time–frequency coefficients into the ridge, i.e., the curve along which the signal energy is locally maximum. However, for the ridge, MSST gives a biased estimate of an IF when dealing with nonlinear frequency modulations; therefore, it is difficult for MSST to estimate IF for nonlinear frequency modulations.

Recently, two methods were proposed to analyze the impulse signals: time-reassigned SST (TSST) [12] and transient-extracting transform (TET) [13]. TSST can achieve a concentrated time–frequency representation for strong FM, whose ridge curves are almost parallel to the frequency axis. TET uses a transient-extracting operator that gives a sharper time–frequency representation than the TSST. However, both the TSST and the TET are not suitable for a signal consisting of FM IFs in the presence of noise.

The high-order SST (HSST) was proposed for IF estimation [14]. However, the computation cost is very high due to the high-order approximation of the FM function.

Synchrosqueezed wavelet transform (SWT) [15] is a new method. It is called an empirical mode decomposition (EMD)-like tool. The anti-noise ability and time–frequency resolution of the SWT are improved on the basis of the WT. After the WT, the corresponding frequency of each scale is given by the derivative of the WT coefficients with respect to time. The SWT greatly improves the energy divergence and time–frequency resolution of the WT. The SWT retains the advantages of both EMD and WT.

The SWT has been widely studied. Yang analyzed the statistical features of the SWT [16]. Reconstruction of the SWT was developed by Meignen [17]. The SWT was extended to the STFT [18], the wave packet transform [19], the S-transform [20], and the curvelet transform [21]. Nevertheless, the SWT and its extensions are suitable only for signals consisting of weak frequency modulated modes [22].

It is shown in [23] that in spite of the improvement in resolution, the SWT fails to identify closely spaced IFs. Their study showed that if the difference in frequency of the two closely spaced IFs is below the sampling rate, the SWT fails to separate them and demands finer sampling of the original signal.

So, in summary, the use of various synchrosqueezing time–frequency representations to IF estimation has multiple essential drawbacks that outweigh its advantage.

In addition, the parameterized TFA methods have been proposed to characterize time–frequency signals using signal-dependent parameters [24]. These TFA methods provide a way to qualitatively analyze the nonstationary signals. However, qualitative analysis cannot be effectively applied to applications such as IF estimation.

The order analysis has been widely applied in nonstationary signal processing and engineering application [25]. In traditional order analysis, the data are measured, using special analogue hardware to sample at a rate proportional to the shaft speed. For the computed order analysis (COA), nonstationary signal and rotational speed are measured at a constant rate, and then use a software to resample the signal at constant angular increments. As a result, the time-domain nonstationary signal is transformed into an angular-domain stationary signal, and characteristic components can be quantitatively evaluated via the order spectrum, generated by the fast Fourier transform. The speed sensors require installation space, extra wiring, delicate calibration, regular maintenance, and therefore have additional cost [26], which seriously limit applications of the COA. For removing restrictions, related to usage of a speed sensors, the IF estimation based COA is developed [27], in which nonstationary instantaneous rotational frequency (RF) is extracted from TFA of nonstationary signals.

However, the RF related IF is difficult to extract in noise environment. In addition, the essential disadvantage of the COA, is errors in the estimation of the shaft angular position and resampled amplitude [28], which affects its accuracy.

The generalized demodulation transform (GDT) is another non-stationary signal processing method, which is proposed by Olhede [29]. The GDT can be used to transform a signal with time-varying frequency component into a signal with constant frequency component. Different to the resample in the COA, the conversion process of the GDT is based on the demodulation operator. Hence, the errors, resulting from estimation of the shaft angular position and resampled amplitude can be avoided [30]. The demodulation operator is calculated via the measured RF or estimated RF. Considering the challenges to separate the overlapped nonstationary multi-component signals, a novel GDT is proposed, based on the GDT [31].

Cheng [32] improved the GDT, and it can be used for multi-component signal decomposition. The iterative GDT is developed to transform multi-frequency components [33]. In the iterative GDT, the true IF curve functions are required, and the multi-frequency curves are transformed into the constant frequency curves. In recent years, the GDT is widely applied in industrial applications, such as fault frequencies detection of bearings and planetary gearboxes under nonstationary conditions [34]. Cheng [35] proposed an envelope order spectrum-based GDT to detect gear fault frequencies. In the proposed method, time-varying fault frequencies are transformed into lines, and are also decomposed into some mono-component. However, the demodulation operator is difficult to obtain without a speed sensor. For example, if the signal component with IF, which is used to calculate the demodulation operator, is polluted by noise, it therefore cannot be extracted from the TFR of the original signal.

Based on the above analysis, the IF estimation technique is important for machineries’ fault frequencies detection under nonstationary conditions. The tachometer-free-based methods have attracted scholars’ attention. Feng proposed [36] an iterative GDT-based energy separation method; the method can be used to process rotational machinery signals with knowledge of true IF dependency. In this method, the GDT is used to separate an interested harmonic, and the harmonic frequency can be extracted. The GDT is applied only one time to the same specific harmonic, and the number of harmonics in a signal defines the number of GDT iterations. These methods are facing the following challenge. In addition to the time-varying IFs, nonstationary signals normally exhibit time-varying instantaneous amplitudes. As a result, frequency components with relatively low amplitudes are easily contaminated by noise, and they therefore cannot be accurately extracted from the TFA by the GDT. The above GDT methods do not consider IFs of components with relatively low amplitudes that are difficult to accurately estimate.

Another challenge is as follows. The GDT transforms a time-varying frequency curve into a constant frequency one, which can be quantitatively characterized by a peak in the spectrum. The transformed frequency curve has a better time–frequency concentration. However, without knowledge of a true IF curve function, the GDT does not produce IF estimations.

In order to address the above-mentioned challenges, the novel IF estimation technology, named multi-generalized demodulation transform (MGDT), is proposed here. The main innovations of the paper are as follows:

1. A novel IF estimation technology is proposed for IF estimation. The proposed novel technology, MGDT, is based on the GDT and employs the novel proposition of multiple adaptive iterations of the GDT for a specific signal component to concentrate blurry TFR energy in a step-wise manner. A theoretical basis of the novel IF estimation technology is created. A novel termination criterion, that could control iterations of the MGDT method, is also proposed. By iteratively and adaptively applying multiple GDT operations to the same specific signal component, the energy of the TFR result should be concentrated in a step-wise manner. The technology does not require a priori knowledge of true IF time dependency in contrast to other advanced IF estimation technologies. This feature is important for industrial applications of the proposed technology.
  The main difference between the proposed MGDT and previously proposed transforms, e.g., [36], is that the MGDT is performing multiple adaptive GDT iterations for the same specific signal component and the previously proposed transforms are performing just one (initial) GDT iteration for the same specific signal component. Other differences are highlighted below. The important feature of the novel MGDT is that every new iteration of the GDT is adaptive, i.e., it is based on previous iteration results.
  Our extensive literature search clearly shows that nobody, in worldwide terms, has previously proposed the main important novelty of the MGDT: an adaptive iterative application of the GDT for the same signal component of a multi-component signal.
2. Novel IF estimation results, obtained by simulation, for four types of AM-FM nonstationary signals under a strong background noise (signal to noise ratio is -5dB): (i) single-component signal with nonstationary amplitude and nonstationary, nonlinear (quadratic) variation of the instantaneous frequency; (ii) multi-component signal with components that have nonstationary amplitudes and nonstationary linear instantaneous frequencies with different chirp rates; (iii) multi-component signal with components that have nonstationary amplitudes and nonstationary, nonlinear (quadratic) instantaneous frequencies with different chirp rates and frequency accelerations; and (iv) multi-component signal with components that have nonstationary amplitudes and nonstationary, nonlinear (cubic) instantaneous frequencies with different chirp rates, frequency accelerations, and the first derivatives of frequency accelerations.
3. Novel experimental IF estimation results for defect frequency of rolling bearings for multiple defect frequency harmonics, using the proposed technology in non-stationary conditions and in conditions of six different levels of a noise interference, including of two levels of a strong noise interference.

The objectives of the research are as follows:

1. To develop, investigate, and create the theoretical basis of the novel IF estimation technology.
2. To perform a novel validation of the proposed technology by simulation for four types of AM-FM nonstationary signals.
3. To perform comparisons of the proposed IF estimation technology with four traditional TFAs technologies by simulation.
4. To perform a novel experimental validation of the proposed technology for IF estimation for multiple harmonics of bearing defect frequency in non-stationary conditions and in conditions of different levels of noise interference, including a strong noise interference.

The rest of the paper is structured as follows. Section 2 presents the developed theoretical bases of the IF estimation technology, including brief review of the GDT, and details of the proposed technology. Section 3 presents novel validation of the proposed method, using four types of the simulated signals, and comparisons of the proposed IF estimation technology with four traditional TFAs technologies by simulation. Section 4 presents a novel experimental validation of the proposed technology for IF estimation for multiple harmonics of a bearing defect frequency in nonstationary conditions and in conditions of different levels of noise interference, including a strong noise interference. Finally, the conclusions are drawn in Section 5.

## 2. Theoretical Bases of the Proposed IF Estimation Technology

In this section, we, firstly, briefly review the GDT technology, and then the novel IF estimation technique, the MGDT, is presented in details.

### 2.1. The GDT Technology

The nonstationary signals, that are AM-FM functions, can be defined as:

(1) $$x\left(t\right)=A\left(t\right)\cos\left(2\pi \int_{0}^{t}f\left(s\right)ds+\emptyset \right)$$

where A(t)>0 is the instantaneous amplitude, f(s)>0 is the IF of the nonstationary signal. ∅ denotes the initial phase. 

In general, the measured signal consists of multiple frequency components, and thus can be expressed by

(2) $$x_{m}\left(t\right)=\sum_{k=1}^{K}A_{k}\left(t\right)\cos\left(2\pi \int_{0}^{t}f_{k}\left(s\right)ds+\emptyset_{k}\right)$$

where *K* is the number of the frequency components.

The analytic signal of signal (1) should be calculated as:

(3) $$\begin{array}{c} x_{A}\left(t\right)=x\left(t\right)+jH\left(x\left(t\right)\right)\\ \approx A\left(t\right)\exp\left(j\left(2\pi \int_{0}^{t}f\left(s\right)ds+\emptyset \right)\right) \end{array}$$

where H(·) represents the Hilbert transform.

Then, the demodulation operator and the modulation operator can be expressed by Equations (4) and (5), respectively.

(4) $$\Phi^{-}\left(t\right)=\exp\left(-j2\pi \left(\int_{0}^{t}f_{d}\left(s\right)ds-f_{c}t\right)\right)$$

(5) $$\Phi^{+}\left(t\right)=\exp\left(j2\pi \left(\int_{0}^{t}f_{d}\left(s\right)ds-f_{c}t\right)\right)$$

where $f_{d}\left(t\right)$ is frequency function; $f_{c}>0$ is a constant frequency.

The demodulation operator is used for demodulation transform and the modulation operator is used for inverse demodulation transform. The demodulated signal can be calculated by multiplying $x_{A}\left(t\right)$ with $\Phi^{-}\left(t\right)$

(6) $$\begin{array}{c} x_{A}^{d}\left(t\right)=x_{A}\left(t\right)\Phi^{-}\left(t\right)=\\ A\left(t\right)\exp\left(j\left(2\pi f_{c}t+2\pi \int_{0}^{t}(f\left(s\right)-f_{d}\left(s\right))ds+\emptyset \right)\right) \end{array}$$

Let $f_{d}\left(t\right)=f\left(t\right)$, the demodulated signal will become a purely AM signal centered around the frequency $f_{c}$. In the GDT technology, the frequency $f_{c}$ is equal to the starting frequency of frequency function f(t).

Similarly, the analytic signal $x_{A}\left(t\right)$ can be calculated by multiplying $x_{A}^{d}\left(t\right)$ with $\Phi^{+}\left(t\right)$ as

(7) $$x_{A}\left(t\right)=x_{A}^{d}\left(t\right)\Phi^{+}\left(t\right)=A\left(t\right)exp\left(j\left(2\pi \int_{0}^{t}f_{d}\left(s\right)ds+\emptyset \right)\right)$$

### 2.2. Novel IF Estimation Technique

The main concept of the IF estimation technique, the MGDT, is to obtain demodulation operator $\Phi^{-}\left(t\right)$ by multiple iterations and step-wise refinement, and it is introduced, based on the mono-component analytic signal, shown in Equation (3).

**Figure 1:**
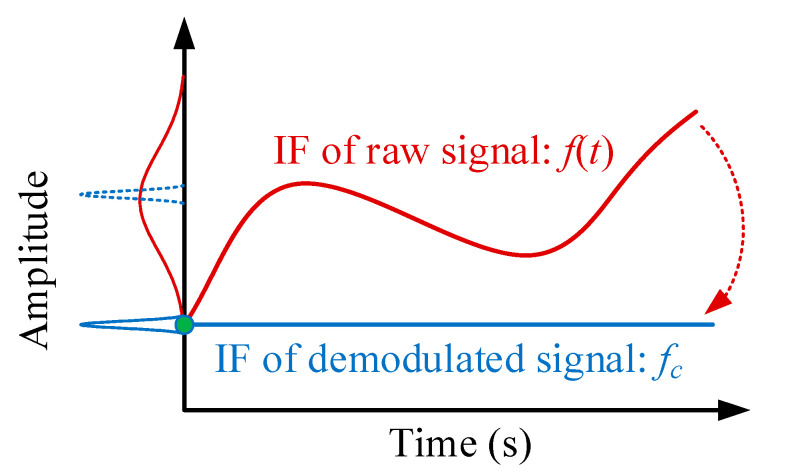
Illustration of the GDT.

Before the demodulation, due to the modulation influence, the spectrum of the signal $x_{A}\left(t\right)$ widely spreads around its IF f(t), as shown in Figure 1. Based on the GDT, with the known demodulation operator $\Phi^{-}\left(t\right)$, the time-varying IF curve can be transformed into a constant frequency $f_{c}$ labelled as a blue line in Figure 1. As a result, its bandwidth is narrow with high energy concentration. Hence, the spectrum of the demodulated signal is more concentrated, than the original spectrum.

**Figure 2:**
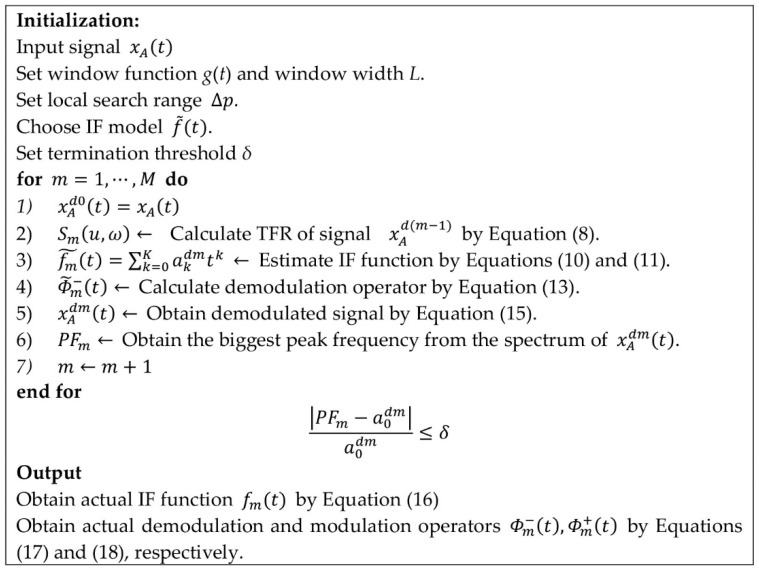
Implementation procedure of IF estimation technique.

Based on relatively higher energy concentration of the demodulated signal, the multiple iteration and stepwise refinement strategies are proposed as follows: (a) roughly estimate the IFs of the original signal from its TFR; (b) calculate the demodulation operator, based on the estimated IF function and roughly demodulate the raw signal; as a result, the bandwidth of the IF curve becomes narrow and time–frequency concentration of the demodulated signal is enhanced and (c) obtain the high-accuracy IFs estimation by the peak searching algorithm. The implementation procedure of the MGDT is presented in Figure 2.

The specifics of the implementation are described in detail as follows

1. IFs and demodulation operator extraction

The rough IFs are firstly extracted from the TFR of the raw signal. The TFR is calculated, based on the STFT. The STFT of the signal $x_{A}\left(t\right)\in L^{2}\left(\mathbb{R}\right)$ with the window function $g\left(t\right)\in L^{2}\left(\mathbb{R}\right)$ can be defined as

(8) $$S\left(u,\omega \right)=\int_{-\infty }^{+\infty }g\left(t-u\right)x_{A}\left(t\right)e^{-i2\pi \omega t}dt$$

The time–frequency resolution of the TFR can be adjusted by the window width, and a wide window results in better frequency resolution, but poor time resolution. An appropriate window width *L* should be determined for obtaining an accurate result.

Then, peak searching algorithm is employed to extract the IFs from the TFR. For improving the robustness against a noise, local maxima detection strategy is developed. The peak searching algorithm is defined as

(9) $$\{\begin{array}{c} IF\left(t_{s},f_{s}\right)=argmax\left[S\left(t,f\right)\right]\\ IF\left(t_{i},f_{i}\right)=arg\underset{\begin{array}{c} t_{i}=t_{s}\pm 1\\ f_{s}-\frac{\Delta p}{2}<f_{i}<f_{s}+\frac{\Delta p}{2} \end{array}}{max}\left[S\left(t_{i},f_{\Delta p}\right)\right]\; \end{array}$$

where *arg max* represents maximize function; Δp is the local search range,$\;IF\left(t_{s},f_{s}\right)$ is position of peak in the whole time–frequency amplitudes; $IF\left(t_{i},f_{i}\right)$ is the output result, *i* = 1, 2, ⋯, *I*. *I* is determined by the window width *L* and the signal length.

For fitting the discrete IFs, there are two function models. The first function model is the polynomial function model, which can be defined as

(10) $$\tilde{f}\left(t\right)=\overset{K}{\underset{k=0}{\sum }}a_{k}t^{k}\;\;\;\;\;\;k\in \left(\mathbb{R}\right)$$

Based on the Weierstrass approximation theorem [37], every continuous function, defined on a closed interval can be uniformly approximated, as closely as need, by a polynomial function. This model is widely used for IF estimation of the chirp signals [38].

The second function model is the Fourier series model, which is defined as

(11) $$\tilde{f}\left(t\right)=a_{0}+\overset{K}{\underset{k=1}{\sum }}\left(a_{k}cos\left(\omega_{k}t\right)+b_{k}sin\left(\omega_{k}t\right)\right)$$

The Fourier series is an expansion of a periodic function in terms of a sum of sines and cosines, and it can break up and recombine an arbitrary periodic function with whatever needed or with a practical accuracy [39]. This model also can be used for IF estimation of the chirp signals [40].

In this paper, the polynomial function model is used to fit discrete IFs. Hence, the first IF curve function is $\tilde{f_{1}}\left(t\right)=\sum_{k=0}^{K}a_{k}^{d1}t^{k}$. The first demodulation and modulation operators can be calculated as

(12) $${\tilde{\Phi }}_{1}^{-}\left(t\right)=exp\left(-j2\pi \left(\int_{0}^{t}\tilde{f_{1}}\left(s\right)ds-a_{0}^{d1}t\right)\right)$$

(13) $${\tilde{\Phi }}_{1}^{+}\left(t\right)=exp\left(j2\pi \left(\int_{0}^{t}\tilde{f_{1}}\left(s\right)ds-a_{0}^{d1}t\right)\right)$$

2. Demodulation and IF accurate calculation

The first demodulation operator ${\tilde{\Phi }}_{1}^{-}\left(t\right)$ is used to demodulate the analytic signal $x_{A}\left(t\right)$. The first demodulated signal $x_{A}^{d1}\left(t\right)$ is calculated as

(14) $$x_{A}^{d1}\left(t\right)=x_{A}\left(t\right){\tilde{\Phi }}_{1}^{-}\left(t\right)=A\left(t\right)\cdot \;exp\left(j\left(2\pi a_{0}^{d1}t+2\pi \left(\int_{0}^{t}\left(f\left(s\right)-\tilde{f_{1}}\left(s\right)\right)ds+\emptyset \right)\right)\right)$$

The first IF curve function $\tilde{f_{1}}\left(t\right)$ is not identical to the actual IF curve function f(t) of the analytic signal. Hence, the spectrum of the demodulated signal $x_{A}^{d1}\left(t\right)$ is not centered around the carrier frequency $a_{0}^{d1}$, but has narrower frequency band than the original spectrum.

The second demodulation operator ${\tilde{\Phi }}_{2}^{-}\left(t\right)$ is calculated for further demodulation, where ${\tilde{\Phi }}_{2}^{-}\left(t\right)$ is obtained by the second IF function $\tilde{f_{2}}\left(t\right)\sum_{k=0}^{K}a_{k}^{d2}t^{k}$, which is extracted from the TFR $S_{2}\left(u,\omega \right)$ of the demodulated signal $x_{A}^{d1}\left(t\right)$.

With the iteration procedure, the actual IF function $f_{m}\left(t\right)$ is calculated as

(15) $$f_{m}\left(t\right)=\overset{K}{\underset{k=1}{\sum }}(a_{k}^{d1}+,\cdots ,a_{k}^{dm})t^{k}+a_{0}^{dm}$$

Furthermore, the actual demodulation and modulation operators can be calculated as

(16) $$\Phi_{m}^{-}\left(t\right)=exp\left(-j2\pi \left(\int_{0}^{t}f_{m}\left(s\right)ds-a_{0}^{dm}t\right)\right)$$

(17) $$\Phi_{m}^{+}\left(t\right)=exp\left(j2\pi \left(\int_{0}^{t}f_{m}\left(s\right)ds-a_{0}^{dm}t\right)\right)$$

Hence, the MGDT result of the analytic signal $x_{A}\left(t\right)$ can be shown as

(18) $$x_{A}^{dm}\left(t\right)=x_{A}\left(t\right)\Phi_{m}^{-}\left(t\right)=A\left(t\right)exp\left(j\left(2\pi a_{0}^{dm}t\right)+\emptyset \right)$$

3. Termination criterion and quantitative evaluation

The iteration can stop if an obvious peak can be captured in the spectrum of the demodulated signal and the biggest peak frequency $PF_{m}$ is approximately equal to the starting frequency $a_{0}^{dm}$ of the IF function $\tilde{f_{m}}\left(t\right)$. Namely,$\;\left|PF_{m}-a_{0}^{dm}\right|/a_{0}^{dm}\leq \delta$. *δ* is the termination threshold. In this paper, *δ* = 0.01. The spectrum of the MGDT signal $x_{A}^{dm}\left(t\right)$ is obtained, using the Fourier transform.

The quantitative indicators of the IF estimation error and transform error are defined to evaluate performance of the MGDT.

The IF estimation error FE is defined as

(19) $$FE=max\left(\frac{\left|f_{m}\left(t\right)-f\left(t\right)\right|}{f\left(t\right)}\right)$$

The transform error TE is defined as

(20) $$TE=\frac{\left|PF_{m}-PF\right|}{PF}$$

where PF is peak frequency in the spectrum of the demodulated signal with theoretical demodulation operator; $PF_{m}$ denotes the biggest peak frequency in the spectrum of the demodulated signal $x_{A}^{dm}\left(t\right)$.

The sensitivity of the MGDT is determined by both the initialization parameters in Figure 2 and noise level. The noise level is the most important parameter. If the signal to noise ratio (SNR) is relatively high, the IFs can be calculated under few iterations. On the contrary, more iterations should be considered under a low SNR.

## 3. Validation of the Proposed IF Estimation Technology via Simulations

### 3.1. Single-Component Non-Stationary Signal Analysis with Nonlinear (Quadratic) FM

For evaluating the performance of the MGDT, a simulated signal, consisting of one frequency component and the Gaussian white noise *n*(*t*) is constructed as

(21) $$x\left(t\right)=\frac{1}{5}e^{2t-4}sin\left(2\pi \left(-36t^{3}+227t^{2}+260t\right)\right)+n\left(t\right)$$

The IF function of the simulated signal is $f\left(t\right)=-108t^{2}+454t+260$; the sampling frequency is 2000 Hz and the time duration is 2 s. It is noted, that this function is not used in the proposed technology; this function is just used for IF error estimation. The SNR is −5 dB, which is calculated as

(22) $$SNR=10log_{10}\left(\frac{p_{x}}{p_{n}}\right)=10log_{10}{\left(\frac{A_{x}}{A_{n}}\right)}^{2}$$

where $p_{x}$ and $A_{x}$ denote power and standard deviation of the noiseless signal, respectively; $p_{n}$ and $A_{x}$ are power and standard deviation of the noise, respectively.

**Figure 3:**
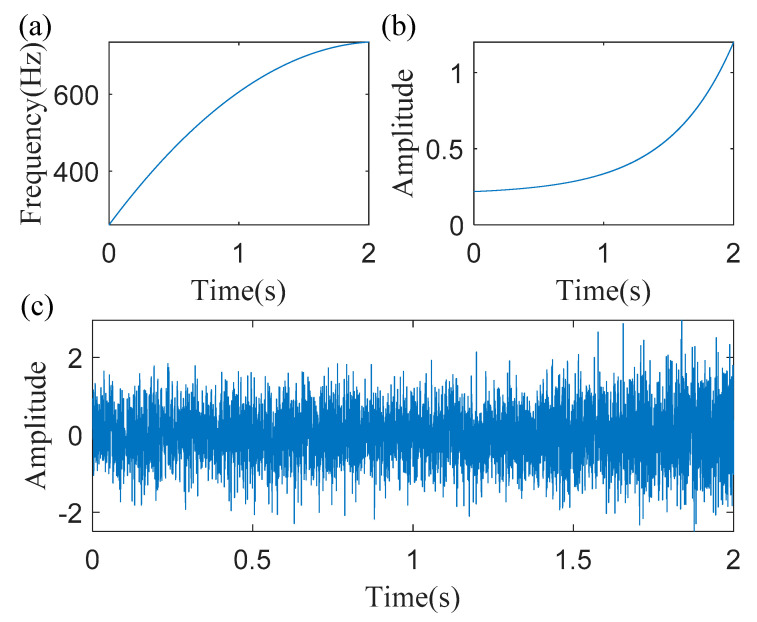
Characteristics of the simulated signal: (**a**) instantaneous frequencies (IF); (**b**) instantaneous amplitude (IA); and (**c**) simulated signal with a noise.

**Figure 4:**
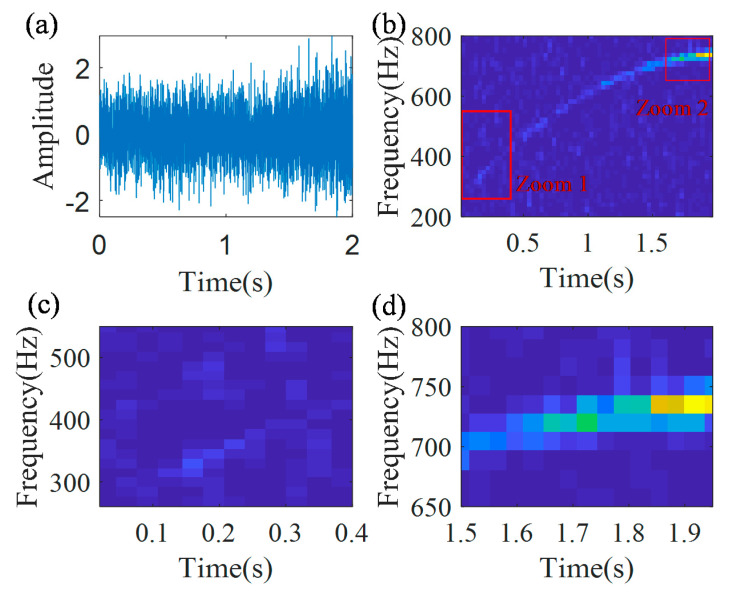
TFR: (**a**) envelope signal; (**b**) TFR; (**c**) local zoom 1; and (**d**) local zoom 2.

Figure 3 shows the simulated signal with a noise, its IF curve and its instantaneous amplitude curve. The instantaneous amplitude of the simulated signal is time-varying, as shown in Figure 3b, i.e., the instantaneous amplitude is relatively low in the initial part of the signal and is relatively high in the final part of the signal. The analytic signal obtained by using the Hilbert transform, is presented in Figure 4a. TFR of the analytic signal, generated by the STFT, and its local zooms are shown in Figure 4b–d, respectively. From the local zooms it can be seen, that the time-frequency ridge in the initial part is much blurry, than that in the final part. The main reason is that the noise pollutes the weak part of the signal.

**Figure 5:**
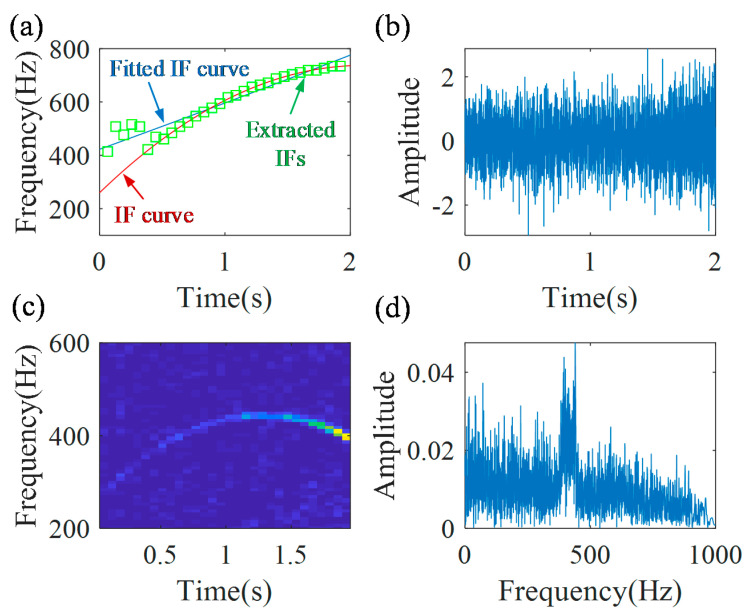
The first demodulation results by the traditional GDT: (**a**) extracted IFs; (**b**) first demodulated signal; (**c**) TFR of first demodulated signal; and (**d**) the spectrum.

The IF estimation technology, the MGDT, is employed to the envelope signal for calculating the IF. Figure 5 shows the first iteration results. Here, the length of the Gaussian window is *L* = 128, 50% overlap, and the local search range is Δp=186Hz. The extracted IFs, fitted IF curve ($\tilde{f_{1}}\left(t\right)=2.96t^{2}+170.64t+422.73$) and the actual IF curve are presented in Figure 5a. It can be found, that due to the inaccurate extraction of IFs in the initial part of the signal, the fitted IF curve has a clear error, which is 62.6%, calculated by Equation (20). Based on Equation (12), the demodulation operator ${\tilde{\Phi }}_{1}^{-}\left(t\right)=exp\left(-j2\pi \left(\int_{0}^{t}\left(2.96s^{2}+170.64s+422.73\right)ds-422.73t\right)\right)$ is calculated. The first demodulated signal is shown in Figure 5b, and its TFR is presented in Figure 5c. The spectrum of the demodulated signal, generated by the Fourier transform, is shown in Figure 5d, from which it can be found, that there is no clear no-smeared peak. Figure 5 also shows the traditional GDT results. It can be found that the traditional GDT is not performing well for this case, i.e., some instantaneous amplitudes are very low and are polluted by a noise.

**Figure 6:**
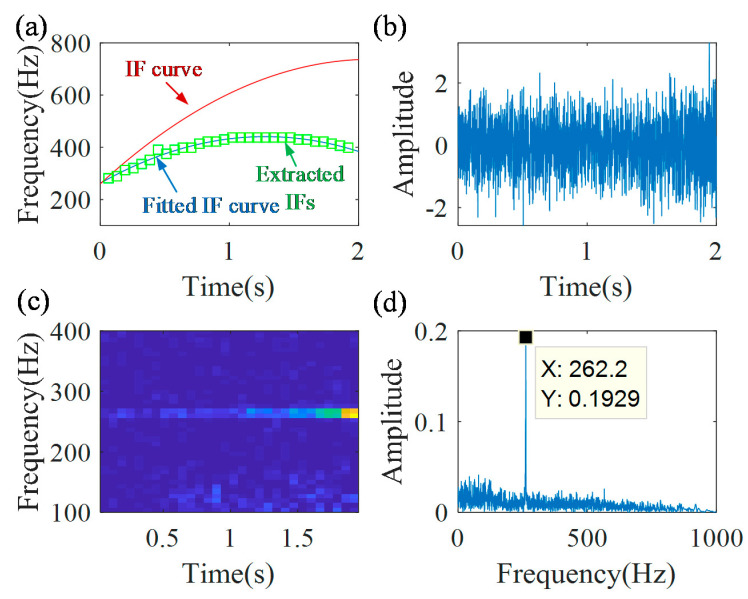
Second demodulation results: (**a**) extracted IFs; (**b**) second demodulated signal; (**c**) TFR of second demodulated signal; and (**d**) the spectrum.

Let continue to the second iteration. The parameters of window function *L* and peak search range ∆*p* are the same, as the parameters in the first iteration procedure. The new IFs are extracted from the TFR of the first demodulated signal and they are plotted in Figure 6a. The fitted IF curve ($\tilde{f_{2}}\left(t\right)=-109.1t^{2}+279.1t+262.4$) is calculated using Equation (10), and it is presented as blue line in Figure 6a. It could be seen a close coincidence between the extracted IFs and the fitted IF curve. Then, the new demodulation operator is calculated ${\tilde{\Phi }}_{2}^{-}\left(t\right)=exp\left(-j2\pi \left(\int_{0}^{t}\left(-109.1s^{2}+279.1s+262.4\right)ds-262.4t\right)\right)$. With the new demodulation operator, the second demodulated signal is obtained, as shown in Figure 6b. Its TFR and spectrum are shown in Figure 6c,d, respectively. There is a constant time–frequency ridge in the TFR. A clear non-smeared peak, whose frequency is 262.2 Hz, can be seen in the spectrum. Based on the termination criterion, i.e., |262.2−262.4|/262.4≤0.01, the iteration process is stopped.

**Figure 7:**
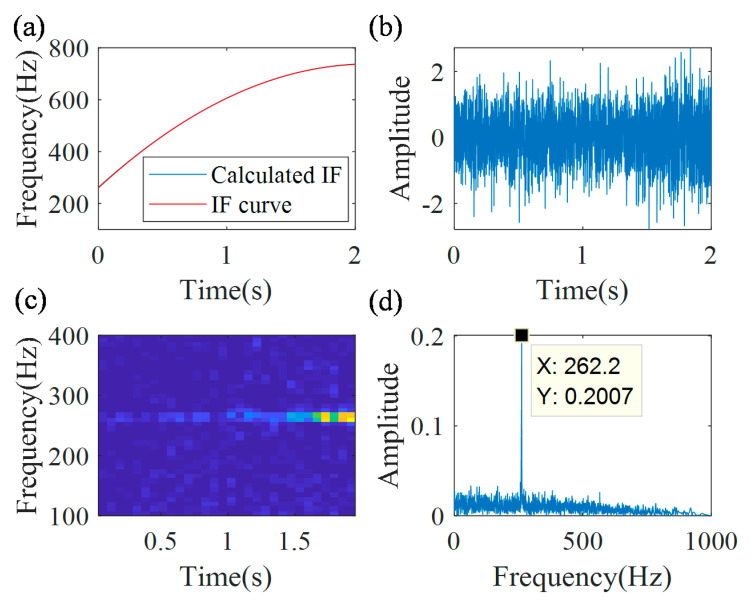
MGDT results: (**a**) calculated IF curve; (**b**) demodulated signal; (**c**) TFR; and (**d**) the spectrum.

The actual IF function $f_{2}\left(t\right)=-106.1t^{2}+449.7t+262.4$ can be calculated based on Equation (15). Figure 7a shows that the calculated IF curve (blue line) coincides with the actual IF curve (red line), and the error is 0.85%. Then, the actual demodulation operator $\Phi_{2}^{-}\left(t\right)=exp\left(-j2\pi \left(\int_{0}^{t}\left(-106.1s^{2}+449.7s\right)ds-262.4t\right)\right)$ is calculated based on Equation (16). With the actual demodulation operator, the demodulated signal $x_{A}^{d}\left(t\right)$ is calculated, as presented in Figure 7b. Its TFR and the spectrum are presented in Figure 7c,d, respectively. As shown in the TFR and the spectrum, the time-varying time–frequency ridge is transformed into a line, which can be quantitatively characterized.

**Figure 8:**
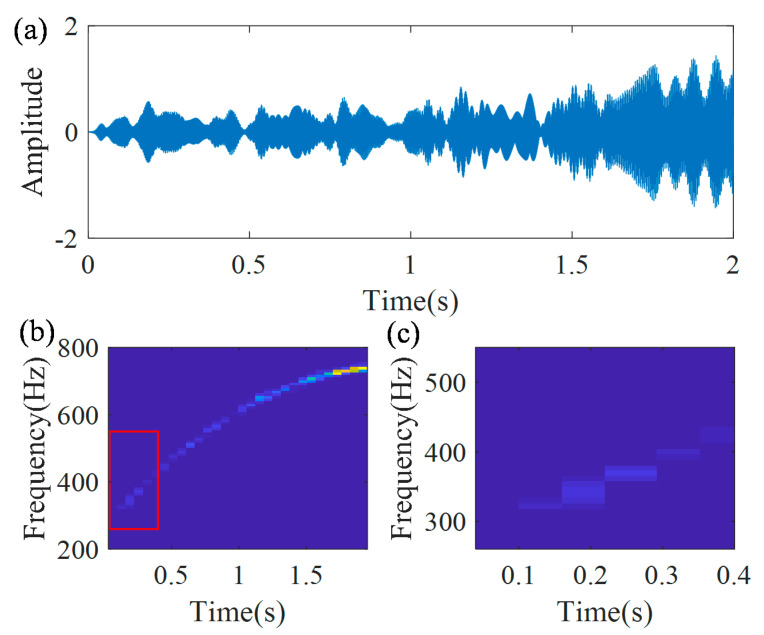
Separated frequency component: (**a**) separated signal; (**b**) TFR; and (**c**) local zoom.

In addition, the frequency component can also be separated from noise, using the inverse MGDT algorithm for avoiding noise interference. The actual modulation operator $\Phi_{2}^{+}\left(t\right)$ can be determined, based on Equation (17). The TFR of the demodulated signal $x_{A}^{d}\left(t\right)$ with one constant time–frequency ridge has well-localized zones of concentration, as shown in Figure 7c. Thus, the band-pass filter is employed to the demodulated signal with the central frequency 262.2 Hz. With the actual modulation operator $\Phi_{2}^{+}\left(t\right)$, the modulation signal $x_{Af}\left(t\right)$ of the filtered component $x_{A}^{df}\left(t\right)$ is calculated as $x_{Af}\left(t\right)=x_{A}^{df}\left(t\right)\Phi_{2}^{+}\left(t\right)$, which is presented in Figure 8a. Figure 8b is the TFR of the modulation signal. Its local zoom is shown in Figure 8c, from which it can be found, that there are no interference components.

For comparison of the MGDT with the traditional TFA methods, the GDT, WT, the SST and the SET are employed.

There are many different families of wavelet functions for various engineering applications. As the proposed MGDT is employing complex analytical signals, it requires a choice of complex wavelet types for comparison purposes [41]. In the present work, the complex Morlet mother wavelet function is selected due to the following main reasons: (i) the Morlet function employs the Gaussian window and, therefore, provides a highly efficient balance between time and frequency resolutions [42]; (ii) the Morlet wavelet is not a sharp edge function; therefore, it minimizes the spectral leakage [43]; (iii) the Morlet wavelet allows the selection of a highly efficient balance between time and frequency resolutions by two parameters: the center frequency and the bandwidth parameter; (iv) the wavelet transform with the Morlet function is computationally efficient due to its link to the fast Fourier transform. For the WT, the wavelet name is “cmor4-2”, the central frequency is 2 Hz, and the bandwidth parameter is 4*s*^2^.

**Figure 9:**
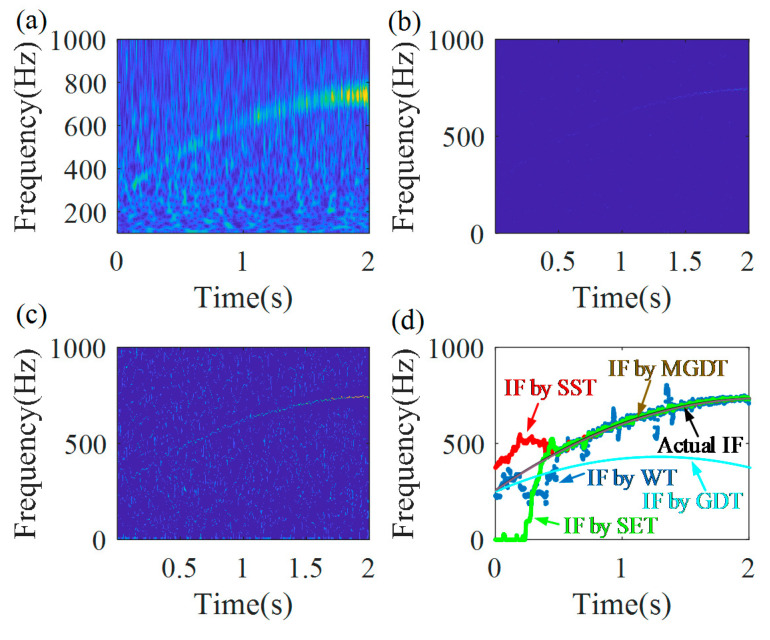
Comparison results: TFRs obtained by (**a**) wavelet transform (WT), (**b**) synchrosqueezing transform (SST) and (**c**) synchro-extracting transform (SET); and (**d**) extracted IFs from TFRs, the actual IF and IF, extracted by the MGDT technology.

For the SST, the window length is 100. For the SET, the window length is 120. The TFRs, calculated by the traditional methods, the WT, the SST, and the SET are shown in Figure 9a–c, respectively.

The IFs are extracted, using the peak search algorithm from the TFRs, and the extracted IFs are presented in Figure 9d. In addition, the actual IF and IF, estimated by the novel MGDT, are also presented in Figure 9d. All peak search parameters are the same, as applied to the proposed MGDT. From Figure 9d, it can be found that the extracted IFs by the GDT, the WT, the SST, and the SET have relatively large errors in the initial duration of the signal. Their total estimation errors are 55.9%, 29.6%, 60.2%, and 113.8%, respectively. The IF estimation error of the GDT, 55.9%, is obtained by use of the traditional GDT just once for the raw signal. The IF estimation errors for different methods are listed in Table 1. It could be seen that the proposed method has a better ability to calculate IFs than the traditional TFA methods, for the simulated signal that features a strong nonlinear FM, a time-variable signal amplitude, and a very noisy environment, the signal to noise ratio is −5dB. It could be also seen from Figure 9d that the main contributions to the estimation errors of the WT, the SST, and the SET are in a very noisy area of a signal, characterized by a low signal amplitude, i.e., in the time slot of (0–0.6) s.

### 3.2. Multi-Component Nonstationary Signal with Linear FM 

For further validation of the IF estimation technology, another multi-component signal with components that have time-varying amplitudes and linear FMs with different chirp rates is simulated on the background of a noise as

(23) $$\begin{array}{c} x\left(t\right)=\frac{1}{5}e^{2t-4}\times \sin\left(2\pi \left(45.4t^{2}+260t\right)\right)+0.9\times \frac{1}{5}e^{2t-4}\times \sin\left(2\pi \left(11.35t^{2}+65t\right)\right)\\ +0.8\times \frac{1}{5}e^{2t-4}\times \sin\left(2\pi \left(2.27t^{2}+13t\right)\right)+n\left(t\right) \end{array}$$

The IF functions of the three components of the simulated signal are $f_{a}\left(t\right)=90.8t+260$, $f_{b}\left(t\right)=22.7t+65$, and $f_{c}\left(t\right)=4.54t+13$, respectively. It is noted that these functions are not used in the proposed technology; these functions are just used for IF error estimations. The sampling frequency is 2000 Hz, signal time duration is 2s, and the SNR is −5 dB.

**Figure 10:**
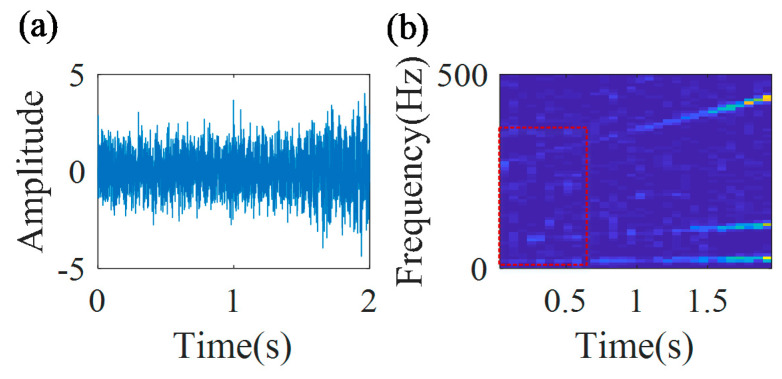
(**a**) Multi-component signal; and (**b**) its TFR.

Figure 10 shows the multi-component signal and the TFR of the envelope signal. From the TFR, it can be found that the time–frequency ridge in the initial part of the signal is much more blurry than that in the final part of the signal.

**Figure 11:**
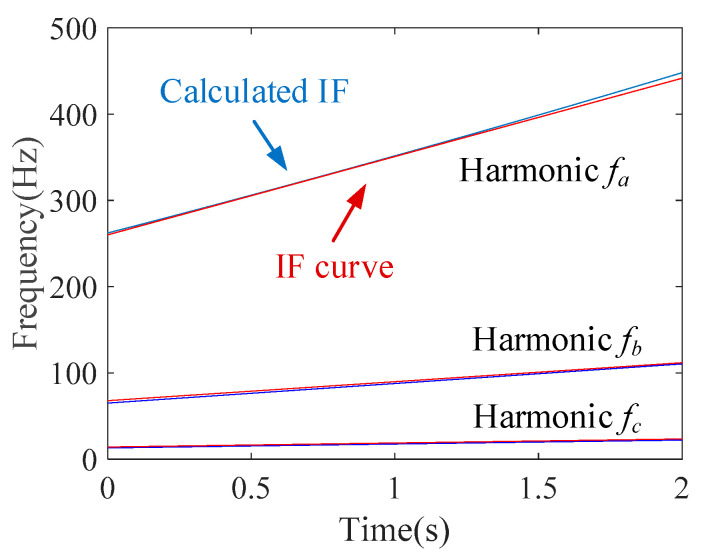
Calculated IF curves.

The IF estimation technology, the MGDT, is employed to the envelope signal for calculating the IF with the highest amplitude. The extracted IF function $f_{a}\left(t\right)=89.1t+256.2$ is obtained. Figure 11 shows that the extracted IF curve (blue line) coincides with the theoretical IF curve (red line), and the estimation error is 1.46%.

Based on the estimated IF $f_{a}\left(t\right)$, the first component can be removed from the raw signal. Then, the MGDT is applied to the signal without the first component, and the IF of the second component is extracted as $f_{b}\left(t\right)=22.2t+66.8$. The estimation error is 2.77%. Then, by removing the second component, the IF of the third component can be extracted as $f_{c}\left(t\right)=4.65t+13.58$, whose estimation error is 4.46%. Hence, the proposed IF estimation technology could be also used for nonstationary multi-component signals with a time-varying amplitude and linear FM.

It should be noted that the amplitudes of components of the signal are different, as shown by Equation (23), and the IF of the component with a higher amplitude is estimated more accurately.

### 3.3. Multi-Component Nonstationary Signal with Nonlinear (Quadratic) FM 

For further validation of the proposed IF estimation technology, another multi-component signal with components that have time-varying amplitudes and nonlinear (quadratic) FMs with different chirp rates and different frequency accelerations is simulated on the background of a noise as

(24) $$\begin{array}{c} x\left(t\right)=\frac{1}{5}e^{2t-4}sin\left(2\pi \left(-7.2t^{3}+45.4t^{2}+260t\right)\right)\\ +0.9\times \frac{1}{5}e^{2t-4}sin\left(2\pi \left(-14.4t^{3}+90.8t^{2}+520t\right)\right)+n\left(t\right)\# \end{array}$$

The IF functions of the two components of the simulated signal are $f_{a}\left(t\right)=-21.6t^{2}+90.8t+260$ and $f_{b}\left(t\right)=-43.2t^{2}+181.6t+520$, respectively. It is noted that these functions are not used in the proposed technology; these functions are just used for IF error estimations. The sampling frequency is 2000 Hz and the time duration is 2 s. The SNR is −5 dB.

**Figure 12:**
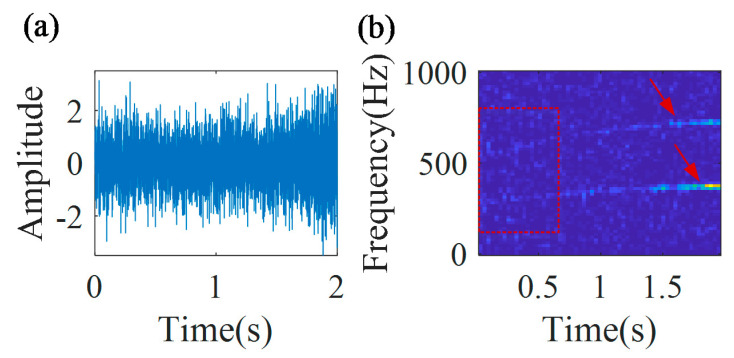
(**a**) Multi-component signal; and (**b**) its TFR.

Figure 12 shows the multi-component signal and the TFR of the envelope signal. From the TFR, it can be found that the time–frequency ridge in the initial part of the signal is much more blurry than that in the final part of the signal.

**Figure 13:**
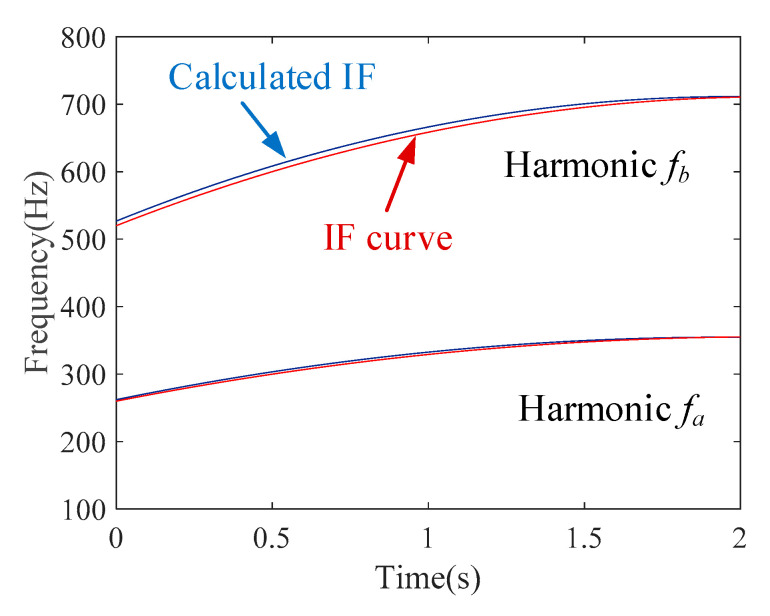
Estimated IF curves.

The IF estimation technology, MGDT, is employed to the signal for calculating the IF with the highest amplitude. The estimated IF function of the first component is $f_{a}\left(t\right)=-24.1t^{2}+94.6t+262.2$. Figure 13 shows that the estimated IF curve (blue line) coincides with the theoretical IF curve (red line), and the estimation error is 1.2%.

Based on the estimated IF $f_{a}\left(t\right)$, the first component can be removed from the raw signal. Then, the MGDT is applied to the signal without the first component, and the IF of the second component is estimated as $f_{b}\left(t\right)=-47.2t^{2}+186.6t+526.8$. The estimation error is 1.4%. Hence, it is validated that the proposed IF estimation technology could be also used for multi-component signals with a time-variable signal amplitude and nonlinear (quadratic) frequency modulations with different chirp rates and different frequency accelerations.

### 3.4. Multi-Component Nonstationary Signal with Nonlinear (Quadratic) FM 

For further validation of the proposed IF estimation technology, another multi-component signal with components that have time-varying amplitudes and nonlinear (cubic) FMs with different chirp rates, different frequency accelerations, and different first derivatives of frequency acceleration is simulated on the background of a noise as

(25) $$\begin{array}{c} x\left(t\right)=\frac{1}{5}e^{2t-4}sin\left(2\pi \left(t^{4}-7.2t^{3}+45.4t^{2}+260t\right)\right)\\ +0.9\times \frac{1}{5}e^{2t-4}sin\left(2\pi \left(2t^{4}-14.4t^{3}+90.8t^{2}+520t\right)\right)+n\left(t\right) \end{array}$$

The IF functions of the simulated signal are $f_{a}\left(t\right)=4t^{3}-21.6t^{2}+90.8t+260$ and $f_{b}\left(t\right)=8t^{3}-43.2t^{2}+181.6t+520$. It is noted that these functions are not used in the proposed technology; these functions are just used for IF error estimations. The sampling frequency is 2000 Hz and the time duration is 2s. The SNR is −5dB.

**Figure 14:**
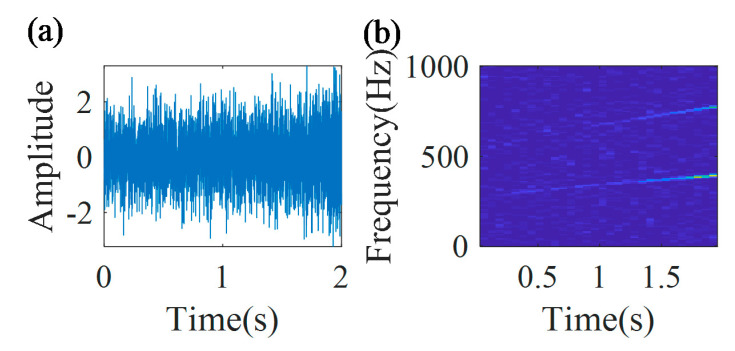
(**a**) Multi-component signal; and (**b**) its TFR.

Figure 14 shows the multi-component signal and the TFR of the envelope signal. From the TFR, it can be found that the time–frequency ridge in the initial part of the signal is much more blurry than that in the final part of the signal.

**Figure 15:**
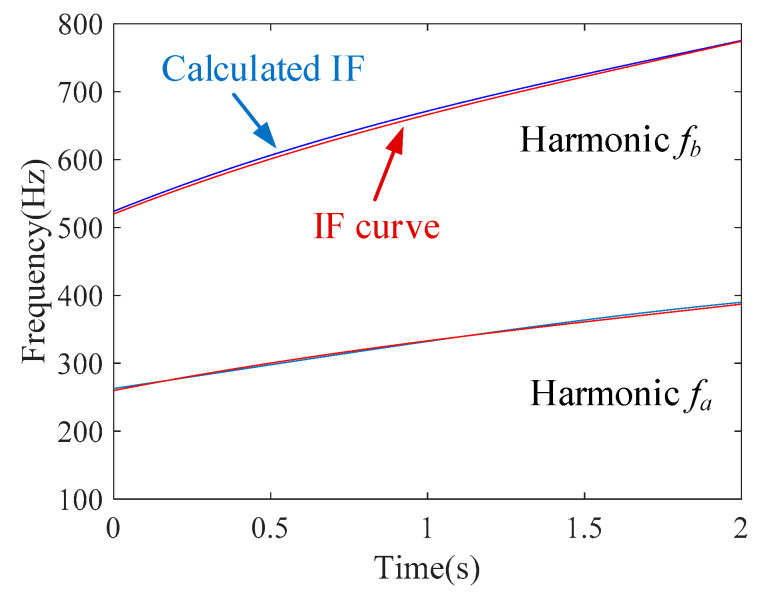
Estimated IF curves and theoretical IF curves.

The IF estimation technology, the MGDT, is employed to the signal for estimating the IFs. Figure 15 shows that the estimated IF curves (blue lines) coincide with the theoretical IF curves (red lines) for both components. The estimation error for the first component is 1.2% and for the second component it is 1.6%.

Hence, it is validated that the proposed IF estimation technology could be also used for multi-component signals with a time-variable signal amplitude and nonlinear (cubic) frequency modulations with different chirp rates, different frequency accelerations, and different first derivatives of frequency acceleration.

The proposed method could be used for the effective online estimation of time-variable linear and nonlinear IFs. The method is designed to estimate IFs from vibration data automatically. Upon receiving, automatically, an IF estimation, related to the first iteration, a termination will be automatically used to determine if the method should continue to iterate or stop further iterations. If an estimation error is lower than a defined termination threshold, the method will stop working. Otherwise, the method will continue to iterate until an estimation error that is lower than a termination threshold is obtained.

The method is computationally fast and provides near real-time estimation results, e.g., the calculation time is 1.7 s for the IF estimation of 2-second vibration data, the frequency range of the STFT is 1000 Hz, the frequency resolution is 7.8 Hz, the overlapping for the STFT is 50%, the sampling frequency is 2000 Hz, and there are two iterations that are employed to achieve the required estimation accuracy.

## 4. Validation of the Proposed IF Estimation Technology via Experimental Trials

In this section, experimental validation of the proposed IF estimation technology for a nonstationary multi-component signal from the rolling element bearing is performed.

The rolling bearing is one of the most widely used mechanical parts in rotating machinery. Its faults are important reasons of shutdowns of machines. For reducing economic losses and avoiding disastrous accidents, novel IF estimations for rolling bearing has become an important topic [44,45,46,47].

The captured nonstationary bearing vibration signal from a faulty bearing is considered for experimental MGDT validation. The main purpose of the validation is to estimate the non-stationary instantaneous bearing defect frequencies for the fundamental harmonic and the higher harmonics, using the proposed IF estimation technology for different levels of noise interference at a very early stage of damage development.

**Figure 16:**
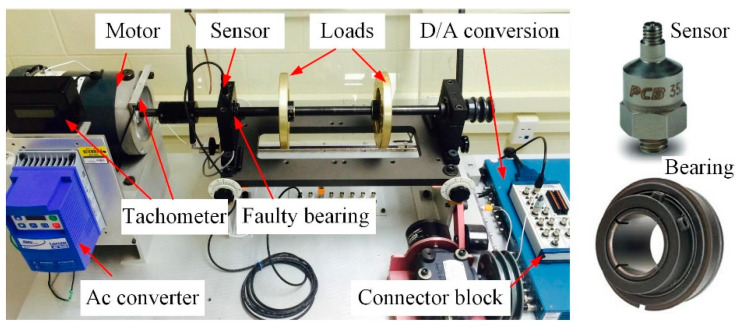
Experimental set-up, a sensor and a bearing.

Figure 16 shows the structural sketch of the experimental set-up. The AC motor (Marathon, Beloit, WI, USA )(3 phase, Hz: 60/50, RPM: 3450/2850, voltage: 208–230/460) is used to drive the steel shaft, which is supported by two bearings (Rexnord, Milwaukee, WI, USA), including a faulty one.

The employed motor has a star connection. In delta connection, each winding will get phase to phase voltage. Alternatively, in star connection, there will be two windings in series across two phases. Hence, motor windings get a higher voltage in delta connection. In star connection, the stator phase voltage is about 58 % of the line voltage. The torque of the motor is directly proportional to the square of the applied voltage. In star connection, the torque of the motor is about 33 % of the maximum rated torque of the motor. The torque delivering capacity of the motor in the delta connection is more than the torque-delivering capacity in the star connection, i.e., torque (star connection) = 33 % of torque (delta connection). Motor vibration amplitudes are propositional to the motor torque. Therefore, it is easier to estimate IFs of narrowband motor components in the delta connection, compared with the star connection.

Two flywheels (SpectraQuest, Richmond, VA, USA) are installed on the shaft to apply load to the system. The RF is measured by a built-in tachometer (SpectraQuest, Richmond, VA, USA) (one pulse per revolution analogue transistor-transistor logic output for DAQ purposes) and the RF can be adjusted by the AC converter.

An accelerometer (PCB, Depew, NY, USA) (PCB 352A60) is mounted on the top surface of the faulty bearing housing by a tapped hole to capture the vibration signal. This miniature accelerometer achieves an extremely high frequency response from 5 Hz to 60 kHz. The sensitivity of the accelerometer is 10 mV/g; electrical filter corner frequency is 45 kHz; electrical filter roll-off is 10 dB/decade; resonant frequency is ≥95 kHz; measurement range is up to 500 g; broadband resolution is 0.002 g rms; nonlinearity is ≤1%; transverse sensitivity is ≤5%; overload limit is 5000 g pk; temperature response is −54 to +121℃; base strain sensitivity is ≤0.05 g/με; excitation voltage is 18 to 30 VDC; constant current excitation is 2 to 20 mA; output impedance is ≤100Ohm; output bias voltage is from 8 to 12 VDC; discharge time constant is from 0.02 to 0.06 s; spectral noise (10 Hz) is 160μg/√Hz; spectral noise (100 Hz) is 40 μg/√Hz; spectral noise (1 kHz) is 15 μg/√Hz; spectral noise (10 kHz) is 10 μg/√Hz; height is 21.6 mm; weight is 6 gm; sensing element is ceramic; size-hex is 3/8in; sensing geometry is shear; housing material is stainless steel; sealing is welded hermetic; electrical connector is 5-44 coaxial; electrical connection position is the top; mounting is integral stud; and mounting thread is 10-32 male. The accelerometer is shown in Figure 16.

The signal collecting system is mainly consisted of a DAQ card (Model: NI-PCI6259, number of channels is 16 differential or 32 single-ended, ADC resolution is 16 bits, single channel maximum is 1.25 MS/s, and multichannel maximal sample rate is 1 MS/s) and the NI-DAQmx software.

The bearing type is ER-10K, number of balls is 8, ball diameter is 8.94 mm, pitch diameter is 33.5 mm, contact angle is 0, and its FCCs of the outer race, inner race, and ball are 3.05, 4.95, and 1.99, respectively. A defect in the form of a 0.1 mm diameter hole is considered in the outer raceway of the bearing; the relative damage size, related to the outer race circumference, is 0.2%. The bearing is shown in Figure 16.

**Figure 17:**
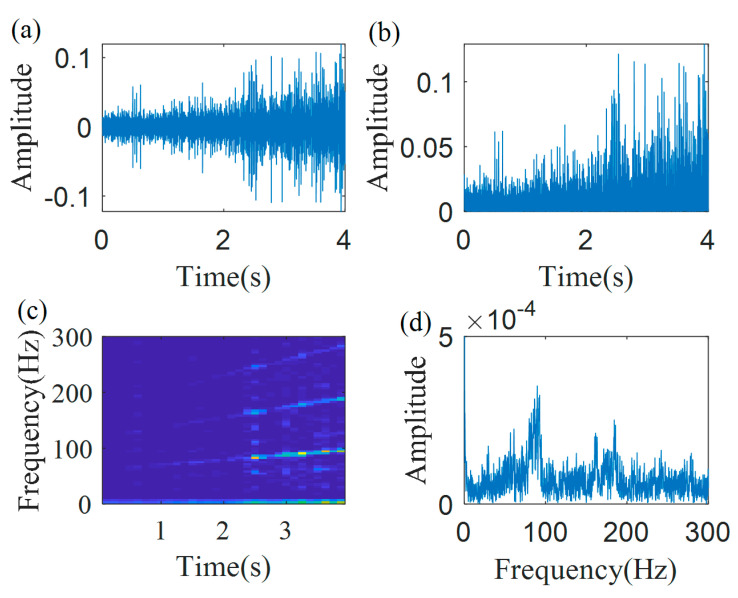
Raw signal: (**a**) signal; (**b**) envelope signal; (**c**) TFR; and (**d**) the spectrum.

First, the raw bearing vibration signal is collected. The collected vibration signal from the bearing with outer race fault is presented in Figure 17a; the estimated from speed sensor RF function is f(t)=2.9t+19.9 Hz; the RF increases from 19.9 to 31.5 Hz over 4 s. The sampling frequency is 48,000 Hz and the time duration is 4 s. It should be noted that speed sensor data are not used for the proposed estimation technology, as the technology does not require a priori knowledge of a true time-varying IF dependency. These data are used only for IF error estimations for the proposed technology.

For selection of the sampling frequency, the following frequencies should be taken into account: the fundamental outer race bearing defect frequency that varies from 60.7 to 96.1 Hz and a frequency band that is related to transient impulses, generated by a bearing outer race local fault. Before the final selection of the sampling frequency, a preliminary assessment of a frequency band that is related to transient impulses, generated by a bearing local outer race fault, was performed by spectral kurtosis [48,49,50], and it was found that this band is (9–12) kHz. So, based on the estimated frequency band, it is clear that the bearing outer race defect frequency is not critical in the selection of the sampling frequency, and the estimated frequency band is critical in sampling frequency selection. In order to avoid aliasing, the sampling frequency is selected as four times the maximum frequency of a frequency band that is related to transient impulses, generated by bearing local faults, i.e., 48 kHz.

Firstly, the proposed technology is validated for IF estimation of the first harmonic of the outer race defect frequency. The envelope signal, shown in Figure 17b, is estimated by the Hilbert transform. Figure 17c,d show the TFR and the spectrum of the envelope signal, respectively. From the TFR, it can be found that due to the noise, the weak IFCFs in the initial duration of the signal are polluted by noise. In addition, because of the time-varying operation condition, the IFCF curve is time-varying and cannot be quantitatively characterized by the classical Fourier spectrum, as shown in Figure 17d.

The proposed IF estimation technology is employed to the envelope signal for obtaining the IFCF. Here, the length of the Gaussian window is *L* = 12000, 50% overlap, the local search range Δp=100 Hz.

The main criterion for window length selection depends on an instantaneous frequency change, characterized by the chirp rate in the case of linear frequency variation and by the chirp rate and the frequency acceleration in the case of quadratic frequency variation, and so on [51,52,53,54].

The employed criterion of window length selection is clearly explained below on the basis of the two main cases of combinations of the instantaneous frequency change $f_{j}\left(t\right)$ of the components j(t) and frequency resolution, defined by a window length.

**Case 1.** The instantaneous frequency change $f_{chj}$ of IF of component $f_{j}\left(t\right)$ during the duration of the selected window is within the frequency limits ($f_{p}-f/2$; $f_{p}+f/2$), defined by the frequency resolution *f* at a particular central frequency $f_{p}$ of a frequency bin of the STFT. The considered case could be viewed as a quasi-stationary case within a window length and no additional errors are provided that are related to STFT estimation for a nonstationary signal, because the time-variable IF is not moving from one frequency bin to another during the selected window length.

**Case 2.** The instantaneous frequency change $f_{chj}$ of IF of component $f_{j}\left(t\right)$ during the duration of the selected window is more than the frequency limits ($f_{p}-f/2$; $f_{p}+f/2$), defined by the frequency resolution f at the particular central frequency $f_{p}$ of a frequency bin.

This case is definitely not a quasi-stationary case and additional errors are provided that are related to STFT estimation for a nonstationary signal, as the time-variable IF is moving from one frequency bin to another during the selected window length, i.e., the IF will be, at least, in two different frequency bins (or even more, depending on the speed of the frequency change) for the selected duration of the window.

Based on the considered two cases, the clear criterion for window selection that we employed is as follows: the instantaneous frequency change $f_{chj}$ of IF of component $f_{j}\left(t\right)$ during the duration of the selected window should be within the frequency limit ($f_{p}-f/2$; $f_{p}+f/2$), defined by the frequency resolution with a “safety factor” within the threshold range (1.5–3) for a “safety factor”, where a safety factor is defined as $S=f/f_{ch}$.

The threshold range (1.5–3) is defined as follows: it is a danger to select a threshold around 1, as an IF still could move from one frequency bin to another during the selected window length. If the selected threshold range is (1.5–3), then the IF will not move from one frequency bin to another during the selected window length. It is possible to have a safety factor of more than 3; however, a drawback of such a big safety factor is that the frequency resolution for this case becomes poorer and this will potentially compromise an IF estimation.

It is discussed here how this criterion was employed for the considered experimental trials.

As the main effort is for estimation, firstly, the IF of the first harmonic of the bearing outer race defect frequency is considered a time variation of the IF of this harmonic, which is 3.05×f(t), where f(t)=2.9t+19.9 f(t) is the time variation of the fundamental rotation frequency, and the coefficient of 3.05 is the FCC of the bearing outer race.

For the selected window length of 12000 (i.e., time duration of this window length is 0.25 s), the instantaneous frequency change f_ch1_ of the IF of the first harmonic of the bearing outer race defect frequency is $f_{chj}$= (0.25*s* x 2.9Hz/*s* x 3.05) = 2.21 Hz and the frequency resolution related to this window length is *f* = (1/0.25Hz) = 4 Hz. So, for this selection of the window, the instantaneous frequency change (i.e., 2.21 Hz) during the duration of the window is within the frequency resolution (i.e., 4 Hz) and, therefore, it is possible to avoid additional errors related to STFT estimation for the first harmonic of the bearing outer race defect frequency. This avoidance is because the time-variable IF is not moving from one frequency bin to another during window length. The “safety factor” for this estimation is *S* = (4 Hz/2.21 Hz) = 1.81 and it is inside the specified threshold range (1.5–3).

It is possible to select a window length, e.g., of 6000 (i.e., the time duration of this window length is 0.125s). In this case, the instantaneous frequency change f_ch1_ of the IF of the first harmonic of the bearing outer race defect frequency is $f_{chj}$= (0.125s x 2.9 Hz/s x 3.05) = 1.11 Hz and the frequency resolution related to this window length is *f* = (1/0.125 Hz) = 8 Hz. So, in this case, the instantaneous frequency change during the duration of the window (i.e., 1.11 Hz) is also within the frequency resolution (8 Hz) and it is also possible to avoid additional errors related to STFT estimation for the first harmonic of the bearing outer race defect frequency.

However, the “safety factor” for this estimation is too big at *S* = (8 Hz/1.11 Hz) = 7.21. A drawback of this big safety is that the frequency resolution for this case (8 Hz) is poorer, compared with window length of 12000 that, potentially, will compromise an IF estimation.

If a longer window length, e.g., 18000, is selected, then the instantaneous frequency change $f_{chj}$ of the IF of the first harmonic of the bearing outer race defect frequency is (0.3755s x 2.9 Hz/*s* x 3.05) = 3.32 Hz, and the frequency resolution related to this window length is *f* = (1/0.375 Hz) = 2.67 Hz. In this case, the instantaneous frequency change during the duration of the window (i.e. 3.32 Hz) is not within the frequency resolution (2.67 Hz) and it is not possible to avoid additional errors related to STFT estimation for the first harmonic of the bearing outer race defect frequency. This non-avoidance is because the time-variable IF is moving from one frequency bin to another during the window length. So, the selection of a longer window length of 18000 is not acceptable.

The same criterion was used for the selection of the window length for simulation trials. For the simulation of two harmonics with nonlinear frequency modulation, the selected window length is 128. For the selected window length of 128 (i.e., the time duration of this window length is 0.064 s), the instantaneous frequency change f_ch1_ of the IF of the first harmonic is 5.72 Hz and the frequency resolution related to this window length is *f* = (1/0.064 Hz) = 15.625 Hz. So, for this selection of the window, the instantaneous frequency change during the duration of the window (i.e., 5.72 Hz) is within the frequency resolution (i.e., 15.625 Hz) and it is possible to avoid additional errors related to STFT estimation for the first harmonic. The “safety factor” for this estimation *S* = (15.625 Hz /5.72 Hz) = 2.73 which is inside the specified threshold range (1.5–3).

So, in order to implement the described criterion for the proposed method, what need to be known, approximately, are the maximum chirp rates for signals with linear variation of the IF and the maximum chirp rates and frequency accelerations for quadratic variation of the IF, etc.

**Figure 18:**
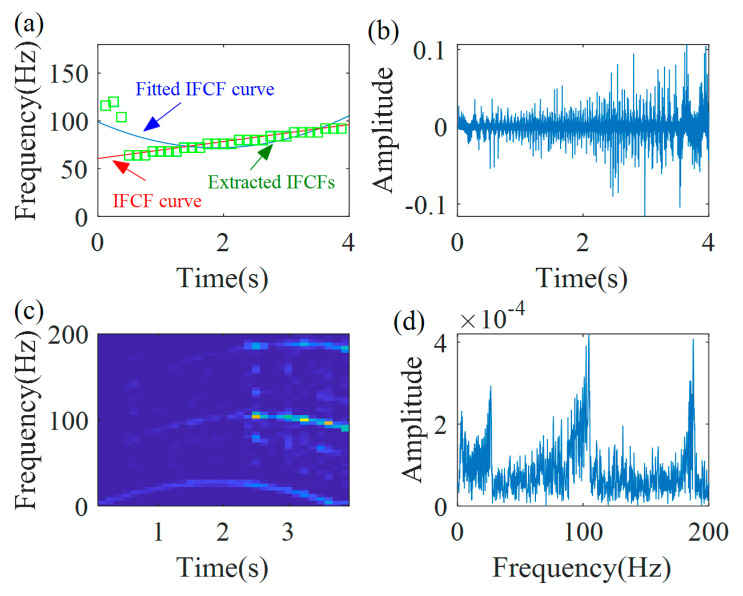
First demodulation results (the traditional GDT): (**a**) extracted IFs; (**b**) demodulated signal; (**c**) TFR; and (**d**) spectrum.

**Figure 19:**
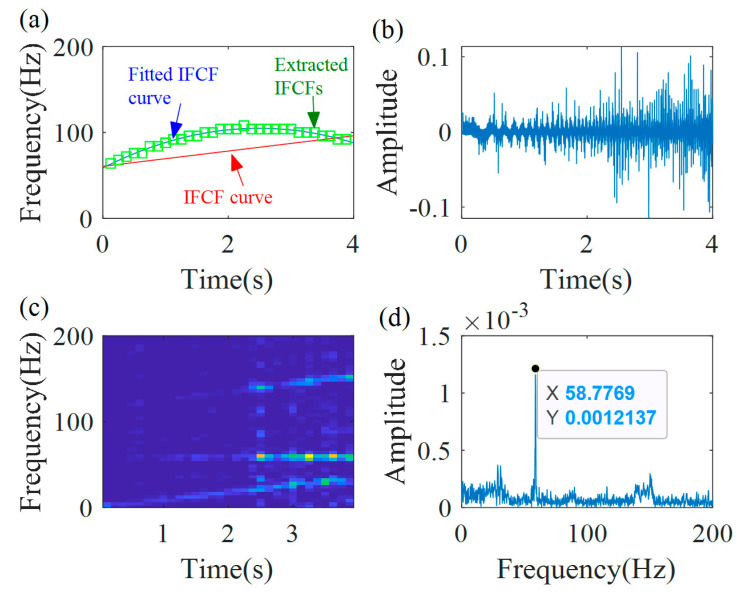
Second iteration demodulation results: (**a**) extracted IFs; (**b**) demodulated signal; (**c**) TFR; and (**d**) spectrum.

Figure 18 shows the first iteration demodulation results. As shown in Figure 18a, the first extracted IFCF has a relatively big error; the error between the first fitted IFCF curve ($\tilde{f_{1}}\left(t\right)=7.6t^{2}-28.6t+99$) and the actual IFCF curve (3.05×f(t)) is 63.1%. A clear peak, without smearing, cannot be captured in the spectrum, as shown in Figure 18d. The second iteration demodulation results are shown in Figure 19. From Figure 19a, it can be found that the extracted IFCF is coincident with the fitted IFCF curve ($\tilde{f_{2}}\left(t\right)=-7.6t^{2}+37.1t+58.8$). There is a clear non-smeared peak in the spectrum, presented in Figure 19d, and the frequency of that peak is very close to the starting frequency of $\tilde{f_{2}}\left(t\right)$.

Based on $\tilde{f_{1}}\left(t\right)$, $\tilde{f_{2}}\left(t\right)$ and Equation (16), the estimated IFCF curve function is $f_{2}\left(t\right)=8.5t+58.8$, and the IF estimation error between the estimated IF function $f_{2}\left(t\right)$ and the obtained one from the speed sensor function 3.05×f(t) is 4.4%. Applying the termination threshold of 0.01, the third iteration is not needed.

**Figure 20:**
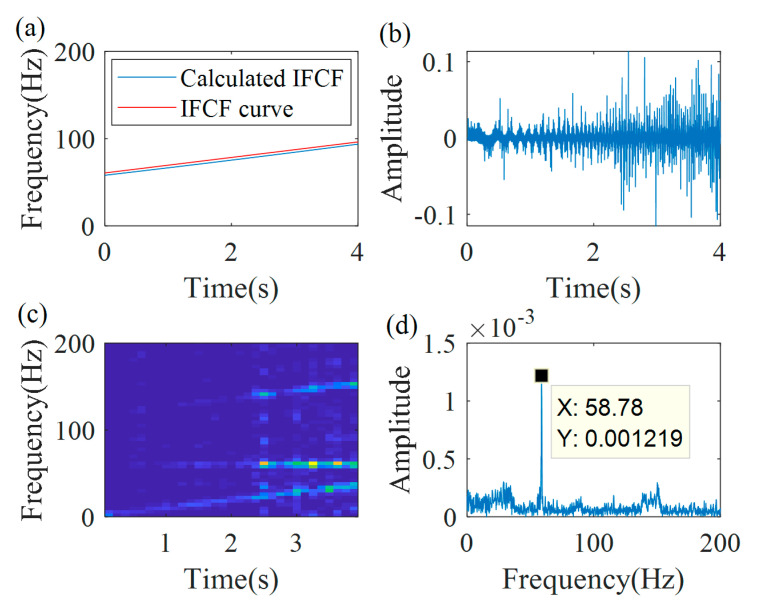
Multiple demodulation results: (**a**) calculated instantaneous fault characteristic frequency (IFCF) curve; (**b**) demodulation signal; (**c**) TFR; and (**d**) the spectrum.

Based on Equation (17), the demodulation operator is estimated as $\Phi_{2}^{-}\left(t\right)=exp\left(-j2\pi \left(\int_{0}^{t}8.5sds-58.8t\right)\right)$. With the obtained demodulation operator, the demodulated signal is estimated and is shown in Figure 20b. Figure 20c,d are the TFR and the spectrum of the demodulated signal. From the TFR, a constant time–frequency curve can be found, and its value is 58.8 Hz, which is a peak frequency in the spectrum, as shown in Figure 20d.

**Figure 21:**
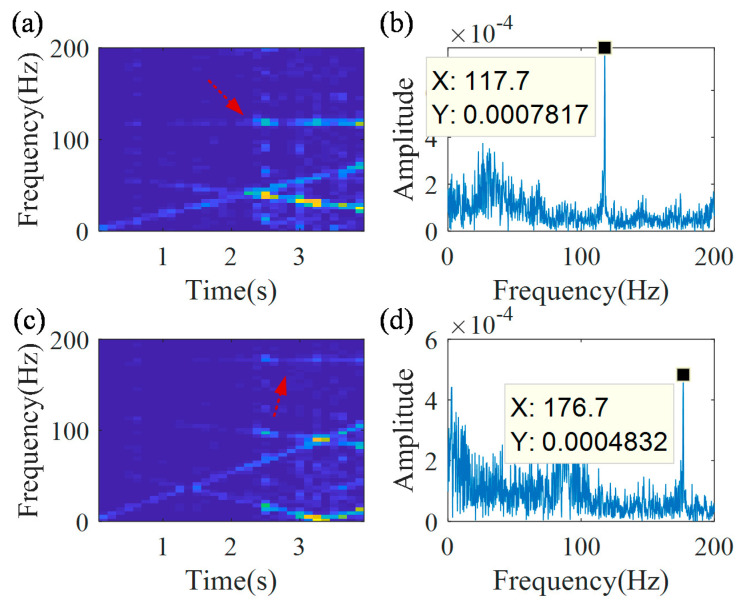
IFCF harmonic-related demodulation results; (**a**) 2nd harmonic-related TFR; (**b**) 2nd harmonic-related spectrum; (**c**) 3rd harmonic-related TFR; and (**d**) 3rd harmonic-related spectrum.

Then, the proposed technology is further validated for estimation of IFs of the second and third harmonics of the outer race defect frequency. Figure 21a,b is the TFR and the spectrum of the demodulated signal, related to the second harmonic. Figure 21c,d is the TFR and the spectrum of the demodulated signal, related to the third harmonic.

A constant time-frequency ridge and a peak can be detected in the TFR and the spectrum of demodulated signal, related to the second harmonic (Figure 21a,b). The peak frequency is 117.7 Hz, which is very close to the value 121.4 Hz, estimated by the speed sensor. Similarly, a constant time-frequency ridge and a peak can be detected in the TFR and the spectrum of the demodulated signal, related to the 3 third harmonic (Figure 21c,d). The peak frequency is 176.7 Hz, which is very close to the value 182.1 Hz, estimated by the speed sensor.

The IF estimation technology is further experimentally validated at different added noise levels. The Gaussian white noise is added to the experimental vibration signal and the SNRs are −4 (strong background interference), 2 (strong background interference), 8, 30, 40, and 50 dB. 

The IF estimation technology is applied to these signals. All technology parameters remain the same. The obtained IFCF estimation errors under different noise levels are listed in Table 2. It can be found that the results have no change under noise levels from 50 to 8 dB compared with the experimental signal without additional noise. The estimation error slightly increases to 5.8% for noise level 2 dB. The estimation error further increases to 7.6%, for noise level −4 dB. Hence, it is experimentally confirmed that a high immunity level against noise is provided by the proposed technology in nonstationary conditions of the very early stage of local bearing damage development, and the relative damage size is 0.2%.

It should be noted that the experimental raw signal has an initial noise level.

As a result of the experimental investigation at different SNRs of the interference, it is obtained that a satisfactory estimation error of 5.8% is achieved at the additional noise level of 2 dB SNR. As experimental vibration data are already contaminated by noise, it is believed that a 5.8% error is achieved, at least, in the range of (0–2 dB) SNR. Thus, the (0–2) dB SNR range is a successful immunity level against interference achieved by the proposed method via experiments. 

It is also shown that the −5 dB SNR is a successful immunity level against noise achieved by the proposed method via simulation, and the simulation error range is (1.2%–4.46%).

In the current experimental setup, the investigated faulty bearing does not slip. In the majority of bearing operation conditions under nominal loads, bearing slippage is insignificant and it is not needed to make any changes to the proposed method. 

However, at high speeds and light loads or at no loads, bearing slippage may be essential. A minimum load must be applied to a bearing in order to avoid slippage. The value of this minimum load increases with speed.

If bearing slippage appears, the fault characteristic periods change and, therefore, the bearing defect frequencies are affected by the slippage. Bearing slippage needs to be taken into account for IF estimation of bearing defect frequencies. For bearing slippage conditions, a novel signal segmentation approach by slicing the bearing vibrations into small realizations (periods), considering simultaneously the relatively small number, e.g., 5–10, of these realizations (i.e., groups of realizations) for IF estimation/fault diagnosis purposes, and then tracking IF estimations/fault diagnosis results via multiple groups has been proposed, developed, investigated, and comprehensively experimentally validated [55,56,57,58,59,60]. This approach takes into account that bearing slippage is not a very fast developing process and, therefore, allows to consider simultaneously 5–10 realizations. It is possible to apply this novel approach for the proposed method for bearing slippage conditions by considering the STFT of a small number of realizations (i.e., groups), applying the method for multiple STFTs of groups, starting with groups characterized by relatively high SNR regions, and tracking the obtained estimation results via multiple groups in order to obtain the overall IF estimation for the whole nonstationary bearing vibration signal.

The proposed method is developed, firstly, as a generic IF estimation method and, secondly, with a specific view of application for IF estimation related to the bearing defect frequencies, for the purpose of bearing fault diagnosis. It is well known that all bearing defect frequencies, i.e., outer race defect frequencies, inner race defect frequencies, rolling element defect frequencies, and cage defect frequencies, are directly proportional to the fundamental rotational frequency and constants of proportionalities which are known as fault characteristic coefficients (FCCs).

Therefore, taking into account these direct proportionalities, it is known that for both stationary and nonstationary conditions of bearing operations and for a simultaneous appearance of multiple bearing local faults (e.g., outer race faults, inner race faults, etc.), the bearing defect frequencies of all harmonics are not intersecting. Even if a rotating shaft with two different rolling element bearings is considered, the bearing defect frequencies of all harmonics are also not intersecting.

Thus, the current version of the proposed IF estimation method is designed for non-intersecting IFs. Other known GDT approaches are also designed for non-intersecting IFs.

Taking into account the nature of the proposed method, the method does not have any limitations on the number of non-intersecting harmonics. It is shown above that the proposed method can successfully estimate multiple IFs for the following cases in a very noisy environment, where the SNR is −5 dB:

- Multi-component signal with three harmonic components with time-variable amplitudes and linear FMs.
- Multi-component signal with two harmonic components with time-variable amplitudes and nonlinear (quadratic) FMs.
- Multi-component signal with two harmonic components with time-variable amplitudes and nonlinear (cubic) FMs.

Therefore, the proposed technology has the potential ability to diagnose multiple bearing faults by IF estimations of bearing defect frequencies, related to local faults of outer race, inner race, cage, and roller elements. As the proposed technology has good immunity against interference, it allows to diagnose early stages of local bearing defects. In the performed experiment, the IF of the outer race defect is successfully estimated at the very early stage of outer race damage development, and the relative damage size is 0.2%.

As the proposed method does not require a priori knowledge about “true IF time dependency”, it could be applied to two scenarios: (i) where bearing defect frequencies are known; and (ii) where bearing defect frequencies are unknown. The last case is very important for industrial applications.

It is possible to apply the proposed method for IF estimations also for induction motors, including inverter-fed induction motors. The proposed method could be used for IF estimations for harmonics related to issues other than bearing faults, such as mechanical faults in induction motors in stationary and nonstationary conditions. In most cases, such an application will be related to IF estimations for variable rotation speed harmonics. In nonstationary conditions of motor operations, these harmonics, normally, have time-variable amplitudes and time-variable nonlinear frequency modulations (pure linear frequency modulations do not often appear in the exploitation of induction motors). Therefore, the three important advantages of the proposed method: the ability of effective IF estimation in conditions of time-variable harmonic amplitude, the ability of effective IF estimation in conditions of time-variable nonlinear frequency modulations, and the ability of the estimation of IF of multiple harmonics, could be exploited for IF estimations for harmonics related to mechanical faults in induction motors.

The effects of inverter harmonics on motor vibration signatures are studied in detail in the literature. It is shown theoretically and experimentally that the vibration signatures, caused by the inverter, are related to the multiple harmonics of the grid supply frequency. It is a possibility that those harmonics will affect the reliability of IF estimations for induction motors as strong inverter-induced harmonics could dominate in vibration spectra and, therefore, will mask weaker harmonics of bearing defect frequencies. However, it is possible to eliminate the influence of inverter-induced harmonics from motor raw vibrations in the same way as gearbox mesh harmonics are removed from gearbox raw vibrations in order to obtain gearbox vibration residual signals, e.g., [48]. In these motor vibration residual signals, masking effects of inverter-induced harmonics will be essentially reduced and, thus, effective IF estimations of motor narrowband harmonics could be performed by the proposed technology.

## 5. Conclusions

The novel IF estimation technology, the multi-generalized demodulation transform (MGDT), is developed and investigated for nonstationary signals, whose nonstationary instantaneous frequencies are unknown and difficult to extract from time–frequency representations in an essentially noisy environment. The theoretical basis of the novel IF estimation technology is created. The main novel feature of the IF estimation technology is the proposition of multiple adaptive iterations of the GDT for the same specific signal component to concentrate blurry TFR energy in a step-wise manner. The important feature of the novel MGDT is that every new iteration of the GDT is adaptive, i.e., it is based on previous iteration results. Our extensive literature search clearly shows that nobody, in worldwide terms, has previously proposed the main important novelty of the MGDT: an adaptive iterative application of the GDT for the same signal component of a multi-component signal.

The proposed IF estimation technology allows to remove the GDT’s restriction of knowledge (a priori or in-advance estimation from a speed sensor) of a true instantaneous frequency that is important for industrial applications of the proposed technology. With the proposed adaptive iterations of the generalized demodulation procedure for the same specific signal component, the energy concentration on the time–frequency ridge is enhanced and a specific signal component with the instantaneous frequency curve, that is polluted by noise, can be accurately estimated.

Novel validation of the proposed technology is successfully performed by the simulation for four types of amplitude- and frequency-modulated nonstationary signals under a strong background noise (signal to noise ratio is −5 dB): (i) single-component signal with a nonstationary amplitude and nonstationary, nonlinear (quadratic) time variation of the instantaneous frequency; (ii) multi-component signal with components that have nonstationary amplitudes and nonstationary, linear instantaneous frequencies with different chirp rates; (iii) multi-component signal with components that have nonstationary amplitudes and nonstationary, nonlinear (quadratic) instantaneous frequencies with different chirp rates and frequency accelerations; and (iv) multi-component signal with components that have nonstationary amplitudes and nonstationary, nonlinear (cubic) instantaneous frequencies with different chirp rates, frequency accelerations, and the first derivatives of frequency accelerations.

The achieved estimation errors for the simulated nonstationary signal in the presence of a strong interference (−5 dB signal to noise ratio) are in the following ranges: (i) (1.46–4.46)% for a multi-component signal with linear frequency modulations and nonstationary amplitudes of components, (ii) (1.2–1.4)% for a multi-component signal with nonlinear (quadratic) frequency modulation and nonstationary variation of amplitudes of components, (iii) (1.2–1.6)% for a multi-component signal with components that have nonstationary amplitudes and nonstationary, nonlinear (cubic) time variations of the instantaneous frequency with different chirp rates, frequency accelerations, and the first derivatives of frequency accelerations, and (iv) 0.85% for a single-component signal with a nonstationary amplitude and nonstationary, nonlinear (quadratic) time variation of the instantaneous frequency.

It is validated by experimental trails that the novel IF estimation technology successfully estimates nonstationary bearing defect frequencies for the first, second, and third harmonics of bearing defect frequencies (without a priori knowledge of true time-varying instantaneous frequency dependency) under time-varying rotation speeds and time-varying amplitudes of these harmonics in the presence of interference and at the very early stage of outer race damage development: the relative local damage size is 0.2%. It is shown that the estimation error for the instantaneous defect characteristic frequency estimation is 4.4%. The instantaneous bearing defect characteristic frequencies of the first, second, and third harmonics, that are polluted by noise in the time–frequency representation, are accurately estimated by the proposed technology.

The estimation errors for nonstationary bearing defect frequencies of the first, second, and third harmonics are experimentally evaluated for six difference levels of added Gaussian noise interference, characterized by a signal to noise ratio (SNR) from 50 to −4 dB, including for two levels of a strong noise interference. It is shown that the estimation errors are unchanged (i.e., staying at a level of 4.4%) under noise levels from 50 to 8 dB, compared with the case without additional noise. The estimation error slightly increases to 5.8% for noise level 2 dB (a strong noise interference) and it further increases to 7.6% for noise level −4 dB (a strong noise interference). These results experimentally confirm a high immunity level against noise delivered by the proposed technology. Based on the experimental validation, for an acceptable IF estimation accuracy, the SNR for the proposed technology should not be lower than (0–2 dB).

The IF estimation technology, the MGDT, is a promising novel concept that could be widely employed for sensor-based vibration instantaneous frequency estimation for nonstationary signals of electromechanical systems in nonstationary operations, in a noisy environment, and without knowledge (a priori or by in-advance estimation from a speed sensor) of true instantaneous frequency variations. The proposed IF estimation technology could be employed for rotating components/machineries such as rolling element bearings, gearboxes, compressors, and induction motors, and could be also effectively employed for other technologies, e.g., low-frequency acoustic non-destructive technologies, e.g., [61,62], motor current signature analysis for induction motors, e.g., [63], ultrasound fault detection technologies, etc.

## Figures and Tables

**Table 1:** IF estimation errors for different methods.

Method	IFs Estimation Error
MGDT	0.85%
GDT	55.9%
WT	29.6%
SST	60.2%
SET	113.8%

**Table 2:** IFCF estimation errors for different noise levels.

SNR	IFCF Estimation Error
No additional noise	4.4%
50 dB	4.4%
40 dB	4.4%
30 dB	4.4%
8 dB	4.4%
2 dB	5.8%
−4 dB	7.6%

## Nomenclature

GDT	Generalized demodulation transform
MGDT	Multi-generalized demodulation transform
IF	Instantaneous frequency
TFR	time–frequency representation
TFA	time–frequency analysis
STFT	Short-time Fourier transform
WT	Wavelet transform
SST	Synchrosqueezing transform
TSST	Time-reassigned SST
MSST	Multi-SST
HSST	High-order SST
TET	Transient-extracting transform
SET	Synchro-extracting transform
COA	Computed order analysis
RF	Rotational frequency
SNR	Signal to noise ratio
IFCF	Instantaneous fault characteristic frequency
FCC	Fault characteristic coefficient
AM-FM	Amplitude-modulated and frequency-modulated
SWT	Synchrosqueezed wavelet transform
EMD	Empirical mode decomposition

## References

[1] Zhao D., Li J., Cheng W., He Z. (2019). Generalized demodulation transform for bearing fault diagnosis under nonstationary conditions and gear noise interferences. Chin. J. Mech. Eng..

[2] Feng Z., Liang M., Chu F. (2013). Recent advances in time–frequency analysis methods for machinery fault diagnosis: A review with application examples. Mech. Syst. Signal Process..

[3] Yu G., Yu M., Xu C. (2017). Synchroextracting transform. IEEE Trans. Ind. Electron..

[4] Iatsenko D., McClintock P.V.E., Stefanovska A. (2015). Linear and synchrosqueezed time–frequency representations revisited: Overview, standards of use, resolution, reconstruction, concentration, and algorithms. Digit. Signal Process..

[5] Li C., Liang M. (2012). A generalized synchrosqueezing transform for enhancing signal time–frequency representation. Signal Process..

[6] Feng Z., Chu F., Zuo M.J. (2011). Time–frequency analysis of time-varying modulated signals based on improved energy separation by iterative generalized demodulation. J. Sound Vib..

[7] Shi J., Liang M., Necsulescu D.S., Guan Y. (2016). Generalized stepwise demodulation transform and synchrosqueezing for time–frequency analysis and bearing fault diagnosis. J. Sound Vib..

[8] Yu G., Wang Z., Zhao P., Li Z. (2019). Local maximum synchrosqueezing transform: An energy-concentrated time–frequency analysis tool. Mech. Syst. Signal Process..

[9] Oberlin T., Meignen S., Perrier V. (2015). Second-order synchrosqueezing transform or invertible reassignment? towards ideal time–frequency representations. IEEE Trans. Signal Process..

[10] Pham D.H., Meignen S. (2017). High-order synchrosqueezing transform for multicomponent signals analysis—with an application to gravitational-wave signal. IEEE Trans. Signal Process..

[11] Yu G., Wang Z., Zhao P. (2018). Multisynchrosqueezing transform. IEEE Trans. Ind. Electron..

[12] He D., Cao H., Wang S., Chen X. (2019). Time-reassigned synchrosqueezing transform: The algorithm and its applications in mechanical signal processing. Mech. Syst. Signal Process..

[13] Yu G. (2020). A concentrated time–frequency analysis tool for bearing fault diagnosis. IEEE Trans. Instrum. Meas..

[14] Hu Y., Tu X., Li F. (2019). High-order synchrosqueezing wavelet transform and application to planetary gearbox fault diagnosis. Mech. Syst. Signal Process..

[15] Daubechies I., Lu J., Wu H.T. (2011). Synchrosqueezed wavelet transforms: An empirical mode decomposition-like tool. Appl. Comput. Harmon. Anal..

[16] Yang H. (2018). Statistical analysis of synchrosqueezed transforms. Appl. Comput. Harmon. Anal..

[17] Meignen S., Oberlin T., McLaughlin S. (2012). A new algorithm for multicomponent signals analysis based on synchroSqueezing: With an application to signal sampling and denoising. IEEE Trans. Signal Process..

[18] Thakur G., Wu H.T. (2011). Synchrosqueezing-based recovery of instantaneous frequency from nonuniform samples. SIAM J. Math. Anal..

[19] Yang H. (2015). Synchrosqueezed wave packet transforms and diffeomorphism based spectral analysis for 1D general mode decompositions. Appl. Comput. Harmon. Anal..

[20] Huang Z.L., Zhang J.Z., Zhao T.H., Sun Y.B. (2016). Synchrosqueezing s-transform and its application in seismic spectral decomposition. IEEE Trans. Geosci. Remote. Sens..

[21] Yang H., Ying L. (2014). Synchrosqueezed curvelet transform for two-dimensional mode decomposition. SIAM J. Math. Anal..

[22] Auger F., Flandrin P., Lin Y.T., McLaughlin S., Meignen S., Oberlin T., Wu H.T. (2013). time–frequency reassignment and synchrosqueezing: An overview. IEEE Signal Process. Mag..

[23] Wu H.T., Flandrin P., Daubechies I. (2011). One or two frequencies? The synchrosqueezing answers. Adv. Adapt. Data Anal..

[24] Yang Y., Peng Z.K., Dong X.J., Zhang W.M., Meng G. (2014). General parameterized time–frequency transform. IEEE Trans. Signal Process..

[25] Guo Y., Liu T.W., Na J., Fung R.F. (2012). Envelope order tracking for fault detection in rolling element bearings. J. Sound Vib..

[26] Shi D., Unsworth P., Gao R. (2006). Sensorless speed measurement of induction motor using hilbert transform and interpolated fast fourier transform. IEEE Trans. Instrum. Meas..

[27] Wang T., Liang M., Li J., Cheng W. (2014). Rolling element bearing fault diagnosis via fault characteristic order (FCO) analysis. Mech. Syst. Signal Process..

[28] Saavedra P., Rodriguez C. (2006). Accurate assessment of computed order tracking. Shock. Vib..

[29] Olhede S., Walden A.T. (2005). A generalized demodulation approach to time–frequency projections for multicomponent signals. Proc. R. Soc. A Math. Phys. Eng. Sci..

[30] Zhao D., Cheng W., Gao R.X., Yan R., Wang P. (2020). Generalized Vold–Kalman filtering for nonstationary compound faults feature extraction of bearing and gear. IEEE Trans. Instrum. Meas..

[31] Yu Z., Sun Y., Jin W. (2016). A novel generalized demodulation approach for multi-component signals. Signal Process..

[32] Cheng J.S., Yang Y., Yu D.J. (2009). Application of the improved generalized demodulation time–frequency analysis method to multi-component signal decomposition. Signal Process..

[33] Chen X., Feng Z. (2016). Iterative generalized time–frequency reassignment for planetary gearbox fault diagnosis under nonstationary conditions. Mech. Syst. Signal Process..

[34] Zhao D., Li J., Cheng W., Wen W. (2016). Compound faults detection of rolling element bearing based on the generalized demodulation algorithm under time-varying rotational speed. J. Sound Vib..

[35] Cheng J.S., Yang Y., Yu D.J. (2010). The envelope order spectrum based on generalized demodulation time–frequency analysis and its application to gear fault diagnosis. Mech. Syst. Signal Process..

[36] Feng Z., Chen X. (2018). Adaptive iterative generalized demodulation for nonstationary complex signal analysis: Principle and application in rotating machinery fault diagnosis. Mech. Syst. Signal Process..

[37] Pérez D., Quintana Y. (2008). A survey on the Weierstrass approximation theorem. Divulg. Mat..

[38] Peng Z., Meng G., Chu F.L., Lang Z.Q., Zhang W.M., Yang Y. (2011). Polynomial Chirplet transform with application to instantaneous frequency estimation. IEEE Trans. Instrum. Meas..

[39] Arfken G. (1985). Mathematical methods for physicists.

[40] Yang Y., Peng Z., Meng G., Zhang W. (2012). Characterize highly oscillating frequency modulation using generalized Warblet transform. Mech. Syst. Signal Process..

[41] Yang S.P., Gu X., Liu Y., Hao R., Li S. (2020). A general multi-objective optimized wavelet filter and its applications in fault diagnosis of wheelset bearings. Mech. Syst. Signal Process..

[42] Gryllias K., Antoniadis I.A. (2013). Estimation of the instantaneous rotation speed using complex shifted Morlet wavelets. Mech. Syst. Signal Process..

[43] Cohen M.X. (2019). A better way to define and describe Morlet wavelets for time–frequency analysis. NeuroImage.

[44] You G., Lv Y., Jiang Y., Yi C. (2020). A novel fault diagnosis scheme for rolling bearing based on convex optimization in synchroextracting Chirplet transform. Sensors.

[45] Zhao D., Wang T., Gao R.X., Chu F. (2019). Signal optimization based generalized demodulation transform for rolling bearing nonstationary fault characteristic extraction. Mech. Syst. Signal Process..

[46] Gao L., Yang Z., Cai L., Wang H., Chen P. (2011). Roller bearing fault diagnosis based on nonlinear redundant lifting wavelet packet analysis. Sensors.

[47] Wu D., Wang J., Wang H., Liu H., Lai L., He T., Xie T. (2020). An automatic bearing fault diagnosis method based on characteristics frequency ratio. Sensors.

[48] Combet F., Gelman L. (2009). Optimal filtering of gear signals for early damage detection based on the spectral kurtosis. Mech. Syst. Signal Process..

[49] Gelman L., Chandra N.H., Kurosz R., Pellicano F., Barbieri M., Zippo A. (2016). Novel spectral kurtosis technology for adaptive vibration condition monitoring of multi-stage gearboxes. Insight-Non-Destructive Test. Cond. Monit..

[50] Gelman L., Petrunin I. (2008). time–frequency higher-order spectra with adjustment to the instantaneous frequency variation. Int. J. Adapt. Control. Signal Process..

[51] Gelman L., Ottley M. (2006). New processing techniques for transient signals with non-linear variation of the instantaneous frequency in time. Mech. Syst. Signal Process..

[52] Gelman L., Petrunin I. (2007). The new multidimensional time/multi-frequency transform for higher order spectral analysis. Multidimens. Syst. Signal Process..

[53] Gelman L. (2007). Adaptive time–frequency transform for non-stationary signals with nonlinear polynomial frequency variation. Mech. Syst. Signal Process..

[54] Gelman L., Petrunin I., Komoda J. (2010). The new chirp-Wigner higher order spectra for transient signals with any known nonlinear frequency variation. Mech. Syst. Signal Process..

[55] Gelman L., Patel T.H., Murray B., Thomson A. (2013). Rolling bearing diagnosis based on the higher order spectra. Int. J. Progn. Health Manag.

[56] Gelman L., Murray B., Patel T.H., Thomson A. (2015). Novel wavelet technology for vibration condition monitoring of rolling element bearings. Insight-Non-Destructive Test. Cond. Monit..

[57] Gelman L., Murray B., Patel T., Thomson A. (2014). Vibration diagnostics of rolling bearings by novel nonlinear non-stationary wavelet bicoherence technology. Eng. Struct..

[58] Gelman L., Murray B., Patel T.H., Thomson A. (2013). Diagnosis of bearings by novel non-linear non-stationary higher order spectra. Insight-Non-Destructive Test. Cond. Monit..

[59] Gelman L., Patel T.H., Persin G., Murray B., Thomson A. (2013). Novel technology based on the spectral kurtosis and wavelet transform for rolling bearing diagnosis. Int. J. Progn. Health Manag..

[60] Gryllias K., Gelman L., Shaw B., Vaidhianathasamy M. (2010). Local damage diagnosis in gearboxes using novel wavelet technology. Insight-Non-Destructive Test. Cond. Monit..

[61] Bouraou N.I., Gelman L.M. (1997). Theoretical bases of the free-oscillation method for acoustical nondestructive testing. J. Acoust. Soc. Am..

[62] Gelman L., Gorpinich S., Thompson C. (2009). Adaptive diagnosis of the bilinear mechanical systems. Mech. Syst. Signal Process..

[63] Ciszewski T., Gelman L., Swędrowski L. (2016). Current-based higher-order spectral covariance as a bearing diagnostic feature for induction motors. Insight-Non-Destructive Test. Cond. Monit..

